# Probabilistic Decision Making with Spikes: From ISI Distributions to Behaviour via Information Gain

**DOI:** 10.1371/journal.pone.0124787

**Published:** 2015-04-29

**Authors:** Javier A. Caballero, Nathan F. Lepora, Kevin N. Gurney

**Affiliations:** 1 Dept of Psychology, University of Sheffield, Sheffield, UK; 2 Faculty of Life Sciences, University of Manchester, Manchester, UK; 3 Dept of Engineering Mathematics, University of Bristol, Bristol, UK; 4 Bristol Robotics Laboratory, University of Bristol and University of the West of England, Bristol, UK; McGill University, CANADA

## Abstract

Computational theories of decision making in the brain usually assume that sensory 'evidence' is accumulated supporting a number of hypotheses, and that the first accumulator to reach threshold triggers a decision in favour of its associated hypothesis. However, the evidence is often assumed to occur as a continuous process whose origins are somewhat abstract, with no direct link to the neural signals - action potentials or 'spikes' - that must ultimately form the substrate for decision making in the brain. Here we introduce a new variant of the well-known multi-hypothesis sequential probability ratio test (MSPRT) for decision making whose evidence observations consist of the basic unit of neural signalling - the inter-spike interval (ISI) - and which is based on a new form of the likelihood function. We dub this mechanism s-MSPRT and show its precise form for a range of realistic ISI distributions with positive support. In this way we show that, at the level of spikes, the refractory period may actually facilitate shorter decision times, and that the mechanism is robust against poor choice of the hypothesized data distribution. We show that s-MSPRT performance is related to the Kullback-Leibler divergence (KLD) or information gain between ISI distributions, through which we are able to link neural signalling to psychophysical observation at the behavioural level. Thus, we find the mean information needed for a decision is constant, thereby offering an account of Hick's law (relating decision time to the number of choices). Further, the mean decision time of s-MSPRT shows a power law dependence on the KLD offering an account of Piéron's law (relating reaction time to stimulus intensity). These results show the foundations for a research programme in which spike train analysis can be made the basis for predictions about behavior in multi-alternative choice tasks.

## Introduction

The decisions we make every day rely on processing continually refreshed streams of uncertain information. This information guides our choices, until some assumed termination criterion is reached, upon which a decision is made [1–6]. Previous influential frameworks have addressed the concerns of uncertainty and time continuity by assuming that new information or *evidence* occurs as continuous stochastic processes [1, 7–10], often mapped at the level of the membrane potential of individual neurons [11–14]. However, most of the ensuing mechanisms are not naturally suited to exploit the statistical structure of the discrete and irregular sequences of action potentials that must ultimately form the substrate for decision making in the brain. As notable exceptions, [15–17] assumed that evidence is sampled from Poisson processes and that statistical inference is conducted upon them. However, their result is founded on the *assumption* that, for Poisson-based spike trains, the evidence was given by counting spikes, and it is not apparent what theoretical foundation might underly this assumption.

Further, there is, as yet, no clear way of dealing with spike trains with arbitrary inter-spike-interval (ISI) statistics, although there has been some interest in log-ISIs as means for sampling for decision formation [18]. Almost all previous models of decision making have worked at a more abstract behavioural level, where the interpretation of evidence is less constrained. Hence, they have either assumed the evidence is Gaussian distributed (*e.g.* [1, 7, 8, 10]) or they have used a general formalism with no distribution specified (*e.g.* [19]). Both views have issues: the negative tail of the Gaussian makes it conceptually inadequate for describing ISIs (or their inverse, the instantaneous firing rate) and better fits are to be had with other skewed probability density functions (pdfs); while the general view does not provide any connection with neural mechanisms, in particular spikes. Typical neural recordings show spike trains which cannot be described by Poisson processes (*e.g.* see [11, 20–27]) and these should be amenable to the same theoretical analysis as their Poisson counterparts. Fortunately, there has been considerable attention paid to identifying the particular pdf that adequately describes variables like these [20, 22, 23, 26, 27]: it is known that the typical distribution of ISIs is positively skewed and has a non-zero mode (maximum). Any neurally grounded account of decision making must accommodate these data.

To address these issues, we introduce here, a general approach to decision making with spikes based only on the assumption that evidence for decisions is conveyed in the distribution of ISIs. Thus, our premise is that the arrival of spikes provides the primary basis of information transfer and that the ISI provides the basic ‘unit of evidence’. The ISI is therefore considered to be an ‘atomic’ time interval that is not linked to some more fundamental, continuous-time process (as is often the case). Our proposal gravitates around a novel variant of the likelihood function which can be applied to any Bayesian or frequentist inference algorithm that uses sequential sampling (for excellent reviews see [9, 28]). With this likelihood we produce a new principled and general formulation of a statistical inference procedure known as the multi-hypothesis sequential probability ratio test [29] (MSPRT). We dub this mechanism *spiking MSPRT* or simply s-MSPRT.

This novel and more general spike-based account allows us to address several previously unanswered questions. This was made possible by investigating several particular cases of the s-MSPRT, each of which assumed a pdf compatible with the distribution of ISIs in existing data [30, 31]. First, noting that the non-zero mode in typical ISI distributions is dictated by an inter-spike refractory period, we ask, what is the advantage of such shape? The refractory period is often seen as drawback for information transmission, but we show it actually furnishes ISIs with additional information for discrimination, thereby facilitating their ability to inform decision making.

Second, given the diversity of s-MSPRT instances now possible, how robust is decision making if the distributions tested-for (the basis of the particular s-MSPRT) are not the same as those of the data being tested? Hitherto, this issue has not been as acute because of the assumption of Gaussian signals and test-distributions. However, as well as parametric differences between test and data distributions, we now have to consider the possibility of a fundamental mismatch between the two, reflecting a potential ‘ignorance’ of the algorithms regarding the actual statistical structure of the task [32]. Here, we show that this issue does not fundamentally compromise the s-MSPRT, thereby revealing a robustness we might expect of a biological mechanism.

Finally we ask, what are the implications of positing a *neural-level* mechanism for decision making like s-MSPRT, at the behavioural level? Our starting point for this analysis is the observation that the mean decision sample size (mean *decision sample* for short) of our algorithms is intimately related to the discrimination information between the distributions of the input data streams, as measured by the Kullback-Leibler divergence (KLD) [33, 34]. Based on this, we demonstrate that the mean total information needed to reach any particular decision is constant and thus conserved. We show how this postulate gives rise to an expression consistent with Hick’s law [35], a well-known psychometric regularity where mean reaction times are shown to approximate a logarithmic function of the number of choices. Lastly, from the same postulate we demonstrate that the mean decision sample of any of our algorithms depends through a power law on the KLD. This bears a strong resemblance to a second regularity known as Piéron’s law [36], where the mean reaction time decreases following a power law as the intensity of the stimulus increases. The KLD between the possible ISI distributions of a sensory neuron should increase for more intense stimuli, we argue, therefore, that our postulate on the conservation of mean total information is a possible explanation for Piéron’s law. We conclude that, the hypothesis that the brain approximates an algorithm like s-MSPRT in its decision making, is consistent with several behavioural phenomena, and that the s-MSPRT provides an explanation for these phenomena grounded in information theory.

Earlier versions of some of these results have been reported in conference abstracts [37, 38] and JC’s PhD dissertation [39].

## Results

### s-MSPRT: a Bayes-based decision mechanism using spikes

We establish an idealised decision making mechanism grounded in the Bayesian approach, which works by sampling a set of spike trains encoding information or *evidence* about a stimulus in a set of parallel data streams or *channels*. The mechanism is a variant of the multi-hypothesis sequential probability ratio test (MSPRT) [29] and must decide which of a set of hypotheses about these data streams is the most probable.

Sequential sampling algorithms usually assume that all data streams are sampled synchronously, and evidence is thereby accumulated for all hypotheses simultaneously. However, our point of departure is to suppose that, in a neural context, evidence is supplied on each channel of a decision mechanism by the arrival of spikes on that channel. Thus, there is a notion of sampling in spike trains grounded in their very construction—that the arrival of a spike within a channel supplies new evidence therein, and conversely, that no new data for the channel is supplied between its spike arrivals. The implication of this is that the notion of synchronous or uniform sampling is lost, because, in general, spike arrivals across channels will be asynchronous. However, it is still possible to establish a sequential sampling scheme that can be used in Bayesian (and indeed, frequentist) inference.

To proceed, consider a decision process over *C* data channels, each one comprised of a neural spike train. Let the process start at *t* ^0^, and let $t_{i}^{0}$, $t_{i}^{1},$ …, $t_{i}^{j},$ … be the sequence of spike arrival times on channel *i*, where $t_{i}^{0}\geq t^{0}$, and $t_{i}^{j}>t_{i}^{j-1}$ for all *j*. We assume the basic unit of data being supplied to the neural decision mechanism is the inter-spike-interval (ISI) defined by $x_{i}(j)=t_{i}^{j}-t_{i}^{j-1}$. The ISIs also serve to define the (non-uniform) data ‘sampling times’ or ‘sampling intervals’ on a *per-channel* basis. There is, therefore, no way of exactly assigning a number of observations consistently across all channels up to a specified time *T*. To define the channel-specific sample size, let $s_{i}(T)=\arg\;\max_{j}\{t_{i}^{j}\leq T\}$. The first and last spikes in the interval [*t* ^0^, *T*] for channel *i* occurred at $t_{i}^{0}$, and $t_{i}^{s_{i}(T)}$ respectively; note that time *t* ^0^ does not necessarily coincide with any spike arrival. Then, the decision process is effective on this channel for an interval $T_{i}=t_{i}^{s_{i}(T)}-t_{i}^{0}$, and there are *s*_*i*_(*T*) ISIs on *i* during this time. Now let **x**_*i*_(*T*) be the set of observations (equivalently ISIs) for channel *i* during the decision process, where **x**_*i*_(*T*) = (*x*_*i*_(1), *x*_*i*_(2),…*x*_*i*_(*s*_*i*_(*T*))). Finally, let *X*(*T*) = {**x**_*k*_(*T*), *k* = 1,…, *C*}. Thus, *X*(*T*) is the entire data set available to the decision mechanism up to time *T*.

We now suppose there are set of *N* hypotheses *H*_*i*_, with *i* ∈ {1,…, *N*}, about the data which we wish to test. At this stage, we retain the most general formalism in which the number of hypotheses, *N*, is not necessarily the same as the number of channels, *C* [40]. Further the hypotheses may concern perceptual interpretations of the data rather than their statistical properties as such [19]. However, subsequently, we specialise to the case where the number of data channels and hypotheses is the same and make more precise the nature of the hypotheses themselves. In general, the hypothesis test requires we compute the posterior probabilities *P*(*H*_*i*_|*X*(*T*)). Using Bayes rule we have

$$\begin{array}{c} P\left(H_{i}|X\left(T\right)\right)=\frac{P\left(X\left(T\right)|H_{i}\right)P\left(H_{i}\right)}{\sum_{k=1}^{N}P\left(X\left(T\right)|H_{k}\right)P\left(H_{k}\right)} \end{array}$$ (1)

where *P*(*H*_*i*_) are the priors for each hypothesis, and *P*(*X*(*T*)|*H*_*i*_) the likelihoods. While our general model allows for a manipulation of the priors to bias choices, for simplicity we hereafter assume that they are all equal. We also take logarithms, thereby transforming fractions to sums

$$\begin{array}{c} \log P\left(H_{i}|X\left(T\right)\right)=\log P\left(X\left(T\right)|H_{i}\right)-\log\sum_{k=1}^{N}P\left(X\left(T\right)|H_{k}\right) \end{array}$$ (2)

Now, following [10, 19], we write this as

$$\begin{array}{c} \log P\left(H_{i}|X\left(T\right)\right)=\log P\left(X\left(T\right)|H_{i}\right)-\log\sum_{k=1}^{N}\exp\left(\log P\left(X\left(T\right)|H_{k}\right)\right) \end{array}$$ (3)

(Lepora and Gurney [19] actually dealt with the negative of the log-posterior which allows the interpretation that the decision is performed by basal ganglia [10]; this is not essential to our exposition here). Then putting *P*_*i*_(*T*) ≡ *P*(*H*_*i*_|*X*(*T*)) and *y*_*i*_(*T*) ≡ log *P*(*X*(*T*)|*H*_*i*_)

$$\begin{array}{c} \log P_{i}\left(T\right)=y_{i}\left(T\right)-\log\sum_{k=1}^{N}\exp\left(y_{k}\left(T\right)\right) \end{array}$$ (4)

It is apparent that a key computation here is the log-likelihood *y*_*i*_(*T*). Assuming independence of data across channels, and no dependence between ISIs within a channel

$$\begin{array}{ccc} y_{i}\left(T\right) & = & \sum_{k=1}^{C}\sum_{j=1}^{s_{k}\left(T\right)}\log p\left(x_{k}\left(j\right)|H_{i}\right)\\ & = & \sum_{j=1}^{s_{i}\left(T\right)}\log p\left(x_{i}\left(j\right)|H_{i}\right)+\sum_{\begin{array}{c} k=1\\ k\neq i \end{array}}^{C}\sum_{m=1}^{s_{k}\left(T\right)}\log p\left(x_{k}\left(m\right)|H_{i}\right) \end{array}$$ (5)

Where *p*(*x*_*k*_(*j*)|*H*_*i*_) is some probability measure applied to *x*_*k*_(*j*) under the hypothesis *H*_*i*_, *e.g.* a probability density (from a pdf) or probability (from a probability mass function). Thus far, we have a very general situation where an arbitrary number of data streams or channels *C* (*e.g.* spike trains from individual neurons) can contribute to any number of hypotheses *N*. However, we now specialise to the case when there is the same number of hypotheses as data streams (*C* = *N*), and each hypothesis *H*_*i*_ takes the following form: that the i.i.d. data *x*_*i*_(*j*) in channel *i* was drawn from a ‘preferred’ pdf f_*_, with mean μ* and standard deviation σ*, while i.i.d. data in other channels *x*_*k*_(*j*), *k* ≠ *i* were drawn from a ‘null’ pdf *f*_0_ with mean *μ*_0_ and standard deviation *σ*_0_ (in general, μ* ≠ *μ*_0_ ≠ σ* ≠ *σ*_0_). Thus

$$\begin{array}{c} y_{i}\left(T\right)=\sum_{j=1}^{s_{i}\left(T\right)}\log f_{*}\left(x_{i}\left(j\right)\right)+\sum_{\begin{array}{c} k=1\\ k\neq i \end{array}}^{N}\sum_{m=1}^{s_{k}\left(T\right)}\log f_{0}\left(x_{k}\left(m\right)\right) \end{array}$$ (6)

The form of the log-likelihood may be simplified by expressing it in terms of probability ratios [10, 19]. To do this we rewrite Eq 6 as

$$\begin{array}{c} y_{i}\left(T\right)=\sum_{j=1}^{s_{i}\left(T\right)}\log f_{*}\left(x_{i}\left(j\right)\right)-\sum_{j=1}^{s_{i}\left(T\right)}\log f_{0}\left(x_{i}\left(j\right)\right)+\sum_{k=1}^{N}\sum_{m=1}^{s_{k}\left(T\right)}\log f_{0}\left(x_{k}\left(m\right)\right) \end{array}$$ (7)

The double sum on the extreme right is hypothesis independent and we denote it by *B*(*T*). Then

$$\begin{array}{c} y_{i}\left(T\right)=\sum_{j=1}^{s_{i}\left(T\right)}\log\frac{f_{*}\left(x_{i}\left(j\right)\right)}{f_{0}\left(x_{i}\left(j\right)\right)}+B\left(T\right) \end{array}$$ (8)

Note that this novel definition of the likelihood, grounded directly in the ISIs of the spike trains, is quite general and may be also used in frequentist sequential sampling methods. It is straightforward to show using Eq 4 that hypothesis independent terms like *B*(*T*) do not alter the posterior, and so we only need consider the first term in Eq 8. We therefore redefine *y*_*i*_(*T*) to be this term, and also introduce some additional notation

$$\begin{array}{c} y_{i}\left(T\right)=\sum_{j=1}^{s_{i}\left(T\right)}L_{i}\left(j\right)\;,\;L_{i}\left(j\right)=\log\frac{f_{*}\left(x_{i}\left(j\right)\right)}{f_{0}\left(x_{i}\left(j\right)\right)} \end{array}$$ (9)

which defines the per-observation ratios *L*_*i*_(*j*). These constitute the ‘evidence contributions’, so that *y*_*i*_(*T*) is the ‘accumulated evidence’.

Then, after substituting Eq 9 in Eq 4, the decision *D*(*T*), at time *T* is:

$$D\left(T\right)=\{\begin{array}{ll} \text{Choose hypothesis}\;i\text{:} & \text{if}\;\text{log}\;P_{i}(T)=\underset{{}_{j\in \left\{1,\ldots ,N\right\}}}{\text{max}}\text{log}\;P_{j}(T)\geq \theta ,\text{at}\;T=T_{D}\\ \text{Continue sampling:} & \text{if}\underset{{}_{j\in \left\{1,\ldots ,N\right\}}}{\text{max}}\text{log}\;P_{j}(T)<\theta \end{array}$$ (10)

**Fig 1:**
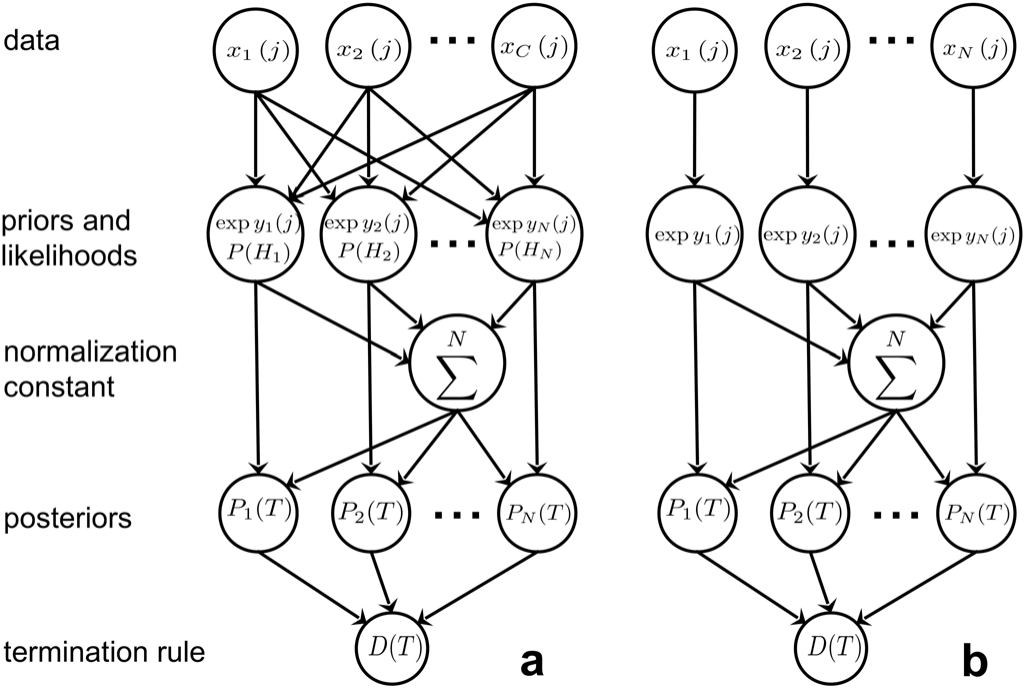
MSPRT in schematic form. Panel a shows the general MSPRT where all the *C* data streams contribute to all of the *N* likelihoods and thus posteriors, which are then evaluated at a termination stage. Panel b only shows the effective components after all simplifications have been applied.

Although an individual threshold can be set per hypothesis [29], for simplicity [10, 16, 17] we assume the single *θ* ∈ [log(1/*N*),0); where the position of *θ* controls the speed-accuracy trade-off. Informally, Eq 10 states that the decision at any given time is: either picking the most salient (likely) hypothesis (*i*), if its decision variable (log *P*_*i*_(*T*)) has surpassed a threshold *θ* at the spike arrival time *T*_*D*_, or continuing to gather data. In what follows, Eq 10 is called the *spiking MSPRT* or simply s-MSPRT. In Fig 1 it is shown in schematic form to illustrate the flow of information through the algorithm.

**Fig 2:**
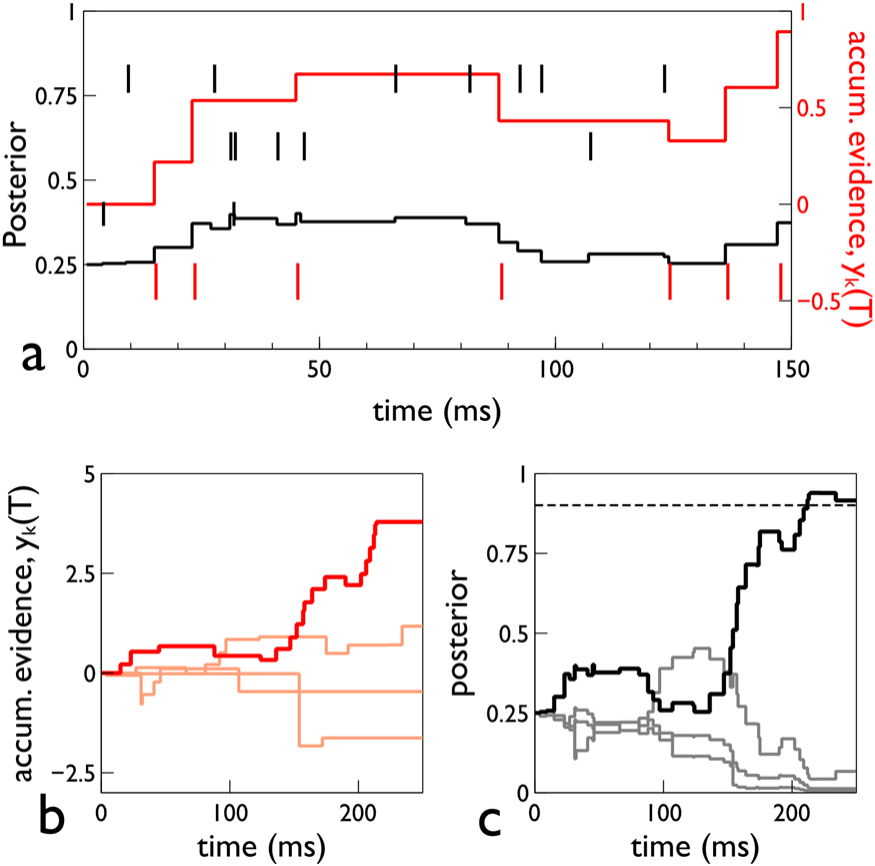
Time course of signals in a single trial of s-MSPRT. The trial is for an s-MSPRT using the gamma distribution, with 4 choices, under the parameterisation set Ω_*IV*_ (see Methods). Panel a, shows the spike rasters of the 4 spike trains as small vertical line markers, with that of the preferred channel in red. This panel also shows the accumulated evidence *y*_*k*_(*T*) as a red line graph. Panel b shows *y*_*k*_(*T*) of all four hypotheses with the preferred hypothesis in bold red. Panel c shows the posteriors with the preferred hypothesis in black and the others in gray. The threshold is shown by the horizontal dashed line, and was chosen to give a 5% error rate.

Fig 2 shows the time course of a single trial in s-MSPRT which serves to illustrate several key points. First, unlike several other instances of the use of MSPRT in neural decision making, s-MSPRT has its sampling grounded in a physically observable process—spike arrivals—which ties it directly to time. Thus, there is no arbitrary synchronous sampling time of some more fundamental continuous-time process, and the decision time emerges naturally in terms of the input data streams. A second observation is that sampling is not uniform; for a given time *T*, there are different numbers of observations per channel, *s*_*i*_(*T*), which depend on the firing in that channel. This means the *y*_*i*_(*T*) are updated at different times, see Fig 2b. Third, while the contribution *y*_*i*_(*T*) is updated only at spike arrival times on channel *i*, the log-posterior log *P*_*i*_(*T*) is updated at the arrival of a spike on *any* channel (see Eq 4 and black line in Fig 2a). Finally, the preceding analysis is quite general; no requirements have been made on the form of the distributions of ISIs (Gaussian or otherwise). In the next two sub-sections, we go on to consider the consequences of using different neurobiologically plausible forms for *f*_0_, f_*_.

### Accumulated evidence with exponentially distributed ISIs is approximately a spike-count

Consider an s-MSPRT with hypotheses that the data is distributed exponentially f_*_(*x*) = λ_*_ exp(−λ_*_ *x*), *f*_0_(*x*) = *λ*_0_ exp(−*λ*_0_ *x*). Here, λ_*_ and *λ*_0_ are mean instantaneous firing rates, defined by the inverse of the respective mean ISI μ*, *μ*_0_. Then, substituting these forms in Eq 9.

$$\begin{array}{ccc} y_{i}\left(T\right) & = & \sum_{j=1}^{s_{i}\left(T\right)}[\log(\frac{\lambda_{*}}{\lambda_{0}})-x_{i}\left(j\right)\left(\lambda_{*}-\lambda_{0}\right)]\\ & = & s_{i}\left(T\right)\log(\frac{\lambda_{*}}{\lambda_{0}})-T_{i}\left(\lambda_{*}-\lambda_{0}\right)\\ & = & s_{i}\left(T\right)\log(\frac{\lambda_{*}}{\lambda_{0}})-T\left(\lambda_{*}-\lambda_{0}\right)+\delta_{i}\left(T\right) \end{array}$$ (11)

where *δ*_*i*_(*T*) = (*T* − *T*_*i*_)(λ_*_ − *λ*_0_) and we have used the fact that summing consecutive ISIs just gives their overall duration (here, *T*_*i*_). The term *T*(λ_*_ − *λ*_0_) is hypothesis independent and can be absorbed into *B*(*T*) in Eq 8. We therefore have

$$\begin{array}{c} y_{i}\left(T\right)=s_{i}\left(T\right)\log(\frac{\lambda_{*}}{\lambda_{0}})+\delta_{i}\left(T\right) \end{array}$$ (12)

or

$$\begin{array}{c} y_{i}\left(T\right)\approx gs_{i}\left(T\right)\text{,}\;\text{with}\;g=\log(\frac{\lambda_{*}}{\lambda_{0}}) \end{array}$$ (13)

and where the ‘error’ term defining the degree of approximation is *δ*_*i*_(*T*).

The right hand side of Eq 13 is a spike count scaled by a ‘gain’ *g* = log(λ_*_/*λ*_0_). This approximation resembles the expression used by Zhang & Bogacz [17] for *y*_*i*_(*T*). The difference between the precise form for *y*_*i*_(*T*) here (Eq 12) and that of [17] stems from them formulating their likelihood function upon discrete Poisson probability mass functions (of spike counts) and us in terms of continuous exponential pdfs (assumed here for ISIs). Nevertheless, up to the approximation in Eq 13, we concur with Zhang & Bogacz that, for Poisson-based spike trains (with exponential ISI distributions), the accumulated evidence *y*_*i*_(*T*) is given by a (gain-multiplied) spike count. However, in deriving their result, Zhang & Bogacz started by assuming that *y*_*i*_(*T*) comprised the spike count, and then deduced and included a separate gain factor. In contrast, we have not assumed any a priori form for the total evidence, and have obtained the integrated form (counts and gain) in Eq 12 directly from the general expression for the sum of individual spike contributions in Eq 8.

To obtain a better understanding of the spike count approximation in Eq 13, use the gain defined there in Eq 12 to obtain

$$\begin{array}{c} y_{i}\left(T\right)=g(s_{i}\left(T\right)+{\hat{s}}_{i}\left(T\right)) \end{array}$$

where ${\hat{s}}_{i}(T)=\delta_{i}(T)/g$ is the error in the spike count. Now consider the spikes immediately prior to, and after *T*. The expectation of the intervals between these spikes and *T* are equal, and their sum is the mean ISI *μ*. Therefore ⟨*T* − *T*_*i*_⟩ = 0.5*μ* and so, for the preferred channel

$$\begin{array}{ccc} \left\langle {\hat{s}}_{i}\left(T\right)\right\rangle & = & \frac{0.5}{g}(1-\frac{\lambda_{0}}{\lambda_{*}})\\ & = & \frac{0.5}{g}(1-e^{-g}) \end{array}$$ (14)

This takes its largest values as *g* → 0, in which case $\langle {\hat{s}}_{i}(T)\rangle \to 0.5$. There is therefore an upper bound on the expected error in the spike count to decision on the preferred channel of 0.5.

### In general accumulated evidence is not given by a spike-count alone

The exponential distribution is not privileged in its relation to Eq 9. Thus, it is possible to obtain a closed form for *L*_*i*_(*j*) for any analytically defined distribution, by substituting a suitably parameterised pair of pdfs for f_*_(*x*) and *f*_0_(*x*) into Eq 9. The results for a range of distributions which may fit ISI data are shown in Table 1. Each distribution has a pair of parameters *ζ*, *η* (not usually the mean and standard deviation of the pdf). Taking two pairs of such parameters ζ_*_, η_*_ and *ζ*_0_, *η*_0_, specifies f_*_(*x*) and *f*_0_(*x*). Substitution in *L*_*i*_(*j*) in Eq 9 yields the expressions in the central column of the table. Each one comprises the sum of a constant *g*_0_, and a sum of products of a coefficient or ‘gain’, *g*_*i*_ (*i* = 1,2), together with a simple function of the variable like log *x*_*i*_(*j*), (log *x*_*i*_(*j*))^2^, *x*_*i*_(*j*)^−1^. Summation over spikes leads, in all instances, to a term like *g*_*i*_ *s*_*k*_(*T*), which is the analogue of the right hand side of Eq 13; that is, it expresses a ‘spike count’ contribution to *y*_*i*_(*T*).

**Table 1:** Analytic expressions for ‘evidence contributions’, *L*_*i*_(*j*) for a range of distributions.

distribution *f*(*x*)	*L*_*i*_(*j*)	gains
exponential *λ* exp(−*λx*)	$g_{0}^{E}-g_{1}^{E}x_{i}(j)$	$g_{0}^{E}=\log\left(\frac{\lambda_{*}}{\lambda_{0}}\right)$ $g_{1}^{E}=\lambda_{*}-\lambda_{0}$
lognormal $\frac{1}{x\Theta \sqrt{2\pi }}\exp\left[\frac{-{\left(\ln x-\kappa \right)}^{2}}{2\Theta^{2}}\right]$	$g_{0}^{L}+g_{1}^{L}{\left[\log x_{i}(j)\right]}^{2}+g_{2}^{L}\log\;x_{i}(j)$	$g_{0}^{L}=\frac{\kappa_{0}^{2}}{2\Theta_{0}^{2}}-\frac{\kappa_{*}^{2}}{2\Theta_{*}^{2}}+\log\left(\frac{\Theta_{0}}{\Theta_{*}}\right)$ $g_{1}^{L}=\frac{1}{2\Theta_{0}^{2}}-\frac{1}{2\Theta_{*}^{2}}$ $g_{2}^{L}=\frac{m_{*}}{\Theta_{*}^{2}}-\frac{m_{0}}{\Theta_{0}^{2}}$
gamma $\frac{x^{\alpha -1}}{\Gamma (k)\beta^{\alpha }}\exp\left(\frac{-x}{\beta }\right)$	$g_{0}^{\gamma }+g_{1}^{\gamma }\log\;x_{i}(j)+g_{2}^{\gamma }x_{i}(j)$	$g_{0}^{\gamma }=\log\left[\frac{\Gamma (\alpha_{0})\beta_{0}^{\alpha_{0}}}{\Gamma (\alpha_{*})\beta_{*}^{\alpha_{*}}}\right]$ $g_{1}^{\gamma }=\alpha_{*}-\alpha_{0}$ $g_{2}^{\gamma }=\frac{1}{\beta_{0}}-\frac{1}{\beta_{*}}$
inverse gamma $\frac{x^{-(\varphi +1)}}{\rho^{\varphi }\Gamma (\varphi )}\text{exp}\left(-\frac{1}{\rho x}\right)$	$g_{0}^{M}+g_{1}^{M}\log\;x_{i}(j)+g_{2}^{M}x_{i}{\left(j\right)}^{-1}$	$g_{0}^{M}=\log\left[\frac{\rho_{0}^{\varphi_{0}}\Gamma \left(\varphi_{0}\right)}{\rho_{*}^{\varphi_{*}}\Gamma \left(\varphi_{*}\right)}\right]$ $g_{1}^{M}=\varphi_{0}-\varphi_{*}$ $g_{2}^{M}=\frac{1}{\rho_{0}}-\frac{1}{\rho_{*}}$
inverse Gaussian ${\left(\frac{\nu }{2\pi x^{3}}\right)}^{1/2}\exp\left[\frac{-\nu {\left(x-\mu \right)}^{2}}{2\mu^{2}x}\right]$	$g_{0}^{S}+g_{1}^{S}x_{i}{\left(j\right)}^{-1}+g_{2}^{S}x_{i}(j)$	$g_{0}^{S}=\frac{1}{2}\log\left(\frac{\nu_{*}}{\nu_{0}}\right)+\frac{\nu_{*}}{\mu_{*}}-\frac{\nu_{0}}{\mu_{0}}$ $g_{1}^{S}=\frac{\nu_{0}-\nu_{*}}{2}$ $g_{2}^{S}=\frac{1}{2}\left(\frac{\nu_{0}}{\mu_{0}}-\frac{\nu_{*}}{\mu_{*}}\right)$

The left hand column shows the canonical functional form of the distribution in terms of its ‘natural’ parameters. The central column is the evidence contribution *L*_*i*_(*j*), and the right hand column contains expressions for the ‘gains’ in terms of such parameters.

**Fig 3:**
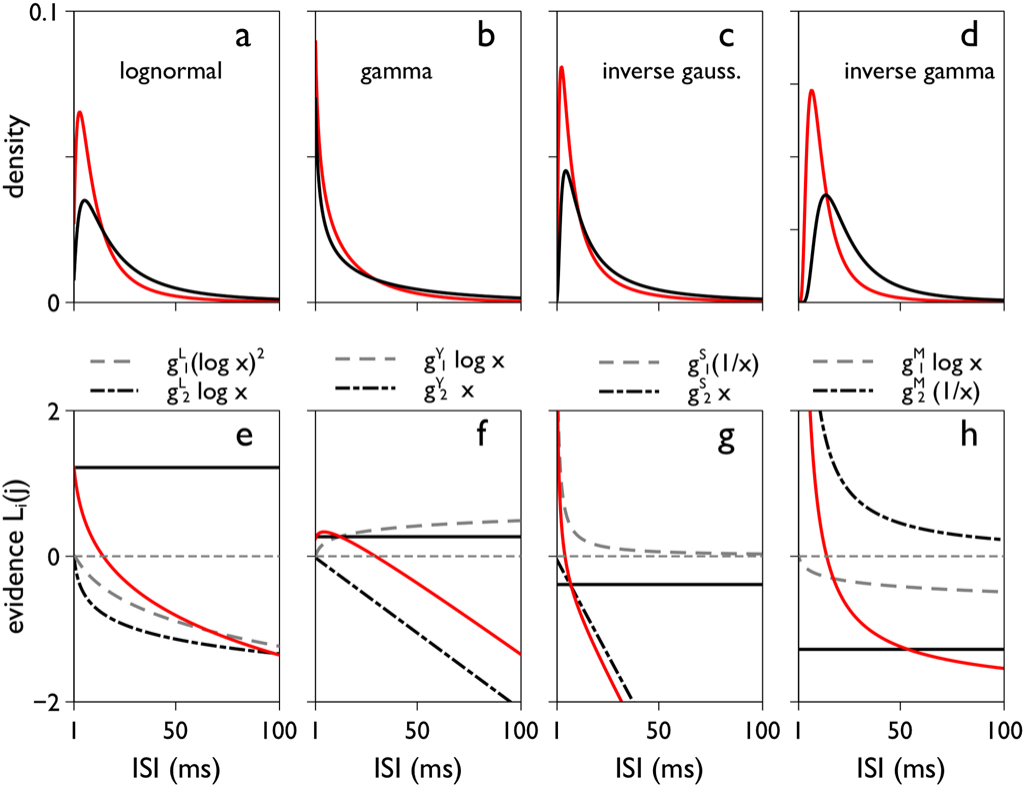
The two-parameter families of pdfs (top) and their ‘evidence contributions’ *L*_*i*_(*j*) (bottom). Panels a-d show the lognormal, gamma, inverse Gaussian, and inverse gamma pdfs respectively, for the independent variance parameter set Ω_*IV*_ (see Methods). The ‘preferred’ and ‘null’ density functions (f_*_, *f*_0_) are in red and black respectively. The plots are for ISIs from 1 to 100 ms. For infinitesimal ISIs, the lognormal, inverse Gaussian and inverse gamma tend to zero; for the the gamma the pdf grows up to a bound as the ISI tends to zero. Panels e-f are the corresponding contributions *L*_*i*_(*j*) to the accumulated ‘evidence’ *y*_*i*_(*T*) (see Eq 9) and the separate components therein (see Table 1). *L*_*i*_(*j*) itself is shown in red, the constant term $g_{0}^{D}$ (*D* = *L*, *γ*, *S*, *M*) by the solid black line, and non-constant terms by dashed-grey and broken-black lines. The horizontal dashed grey line indicates 0 on the *y*-axis.

However, there are other terms in *L*_*i*_(*j*) which depend on *x*_*i*_(*j*) and which can make a substantial contribution to the development of *y*_*i*_(*T*). This is demonstrated in Fig 3 for a particular set of biologically plausible pdfs and parameterisations, Ω_*IV*_, described in the Methods. Fig 3a–3d, shows the pdfs specified by Ω_*IV*_. In the corresponding panels e-h below, *L*_*i*_(*j*) is shown per pdf as a function of *x*_*i*_(*j*) (solid red line) as well as the functions for its contributory terms. The term *g*_0_ is constant (solid black line), the spike count contribution and other non-linear terms are functions of *x*_*i*_(*j*) (dashed and broken gray/black lines). There is clearly a wide variation in non-constant contributions to *L*_*i*_(*j*). Most notably, the terms linear in *x*_*i*_(*j*) in the gamma and inverse Gaussian, have an apparently disproportionate effect on *L*_*i*_(*j*). However, as noted earlier in connection with the exponential distribution, when summed over spikes, such terms give an expression which is approximately *gT* (for some gain *g*). If these terms were identically equal to *gT*, they may be absorbed into the constant term *B*(*T*) in Eq 8, and have a null effect on the posterior. Thus, assuming the terms linear in *x*_*i*_(*j*) are a good approximation to *gT*, they will have a very limited influence on the outcome of a decision.

### s-MSPRT has a ‘regular’ MSPRT counterpart for all ISI distributions

It is instructive to compare the s-MSPRT with a counterpart ‘regular’ MSPRT with temporally *uniform*-sampling; that is, in contrast to neural spike trains, observations are drawn simultaneously for all channels every time interval *δt* (MSPRT as in [10, 19, 29]). We denote this uniform-sampling variant, u-MSPRT. Note this alternative form, like its s-MSPRT counterpart, is not supposed to be based on observations from an underlying continuous process. Rather, u-MSPRT relies on a fundamentally discrete, sequential process with uniform inter-observation time, but is abstract and does not assume any explicit representation of spike arrivals. Its sole purpose is to provide a ‘bridge’ from the s-MSPRT to the more usual, uniformly sampled decision processes (which may well assume a continuous time foundation). In this scheme, the distribution-independent formalism developed above is preserved almost in its entirety, with *x*_*k*_(*j*) interpreted as the *j* ^th^ observation on channel *k*, drawn from one of the pdfs describing ISIs. Thus, Table 1 applies for all such u-MSPRT, which therefore extends previous results describing the specific form of MSPRT for Gaussian inputs only [10].

In all u-MSPRT variants, the expressions contributing to *y*_*i*_(*T*) in Eq 9 now refer to sums up to observation *s*(*T*) for any channel (instead of *s*_*i*_(*T*)) and *T* = *s*(*T*)*δt* (*T*_*i*_ = *T*, for all *i*). With likelihoods and posteriors thus updated simultaneously for every hypothesis, every *δt*, u-MSPRT also takes the form in Eq 10 and has the structure in Fig 1.

We now turn to the comparison between s- and u-MSPRT. For both variants, the decision time is governed by the *decision sample*—the number of observations required to reach the threshold. For a given decision, in s-MSPRT the decision sample will depend on the hypothesis which has reached threshold. On average, when μ* < *μ*_0_, there will be more observations in a preferred channel than in one of the null channels; this is observed in cortical sites that supply evidence for decision formation, like the middle-temporal visual area (MT) [30] or the primary somatosensory cortex [24]. We report the expected decision sample with respect to an equivalent number of preferred channel observations, $\left\langle s_{s}^{*}\right\rangle$, irrespective of the hypothesis crossing the threshold. For u-MSPRT, all channels are sampled equally frequently but we will, nevertheless, denote the expected decision sample with respect to the preferred channel, $\left\langle s_{u}^{*}\right\rangle$, in order to facilitate comparison between u- and s-MSPRT.

For s-MSPRT, the decision time $T_{D}^{s}$ is the sum of an integer number of observations, $s_{i}(T_{D}^{s})$, and a ‘residual time’ comprising the time from *t* ^0^ to first spike arrival at $t_{i}^{0}$. For correct decisions, the expected value of *x*_*i*_(0) is 0.5μ*. Thus, the expected decision times for each type of MSPRT are given by

$$\begin{array}{ccc} \left\langle T_{D}^{s}\right\rangle & = & \left(\left\langle s_{s}^{*}\right\rangle +0.5\right)\mu^{*}\\ \left\langle T_{D}^{u}\right\rangle & = & \langle s_{u}^{*}\rangle \delta t \end{array}$$ (15)

Notwithstanding the simple formal relations above, the interpretation of decision making by these algorithms, in terms of an overall *decision time*, is rather subtle. For s-MSPRT, observations are explicitly determined by spike arrivals, and we will ultimately need to know whether we are we dealing with single or multiple spike trains and, if the latter, how these combine to make ISI-pdfs for algorithm input. These questions are taken up again in the Discussion but, in all subsequent results, we consider processing of an individual afferent spike train as the ‘unit of decision making’. All the analyses of ISI statistics described above are, therefore, directly applicable. Further, in previous application of uniform-sampled MSPRT to neurobiological decision making, the parameters were chosen to allow *behaviourally* appropriate decision times [10]. Here, however we wish to examine decision making at the level of spike trains and *neural* processing which requires a different approach relating observations in u-MSPRT to ISIs.

Given these issues, we will report results (for both u- *and* s-MSPRT) in terms of the decision sample rather than overall decision times. In particular, we can ask whether the decision samples for each of u- and s-MSPRT are similar. Given the mechanistic difference between s- and u-MSPRT in terms of a homogeneous versus heterogeneous channel sampling rate, it is not clear *a priori* whether this is necessarily the case. However, if they are indeed similar, then notwithstanding the problems with interpreting decision times, it is instructive to see what choice of sampling period *δt* would ensure similar decision times. The investigation to answer these questions is largely empirical, but we supply heuristic analysis to give insight into the outcomes.

**Fig 4:**
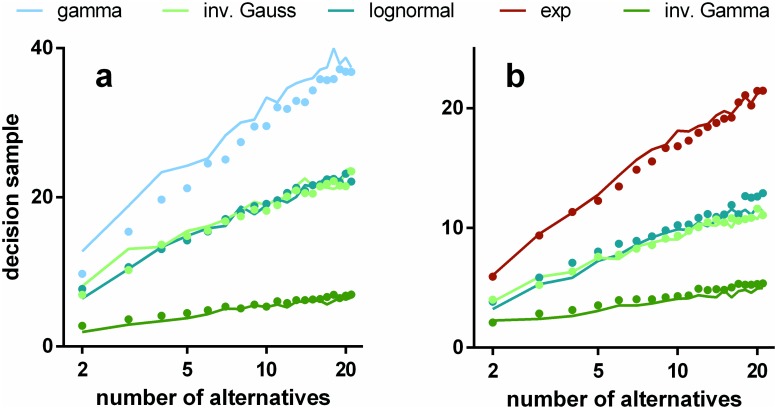
Mean decision samples against number of choices for a range of pdfs, parameter sets, and mechanisms. Each panel shows mean decision sample as a function of the number of choices for a range of pdfs (see legend) and for the two alternative mechanisms: s-MSPRT (solid lines) and u-MSPRT (solid circles). Panels a and b are for the parameter sets Ω_*IV*_ and Ω_*FV*_ respectively (see Methods). In the case of Ω_*FV*_, the gamma and exponential distributions are identical and so not reported separately. All data points are the mean of 950 correct, out of 1000 total trials (see text for inverse gamma based s-MSPRT). Error bars are omitted for clarity and are small; the standard error of the mean is typically 2% of the mean.

We ran simulations of s-MSPRT as a function of the number of choices or hypotheses, *N*, for a range of pdfs, and the two parameter sets Ω_*IV*_, Ω_*FV*_ (see Methods). All the simulation results use trials with an error rate of 5% obtained by iteratively seeking a threshold that satisfied this criterion. Every data point for a particular number of alternatives is the mean over 950 correct, out of 1000 total trials (982 correct for the inverse gamma based s-MSPRT at *N* = 2 with Ω_*FV*_ in Fig 4 which is at an error rate of 1.8% as it was not possible to reliably achieve a 5% error here; this decision task would appear to be too easy to be compromised to this extent). The large trial numbers ensure that estimation of the error rate during threshold determination is sufficiently accurate. For the simulations in this section, the ISIs are drawn from the distributions, f_*_ for the preferred channel, and *f*_0_ for the null channels.

The decision samples $\left\langle s_{s}^{*}\right\rangle$ are estimated in Fig 4 (solid lines). We can consistently interpret these results as decision times because μ* = 16.5 ms throughout. Then, assuming only the single preferred afferent spike stream, Eq 15 implies that 10 observations translates to 165 ms. The solid symbols in Fig 4 show the comparable decision samples $\left\langle s_{u}^{*}\right\rangle$ for u-MSPRT. There is clearly a very close correspondence between the two sets of mean decision samples across a range of conditions; that is, $\left\langle s_{u}^{*}\rangle \approx \langle s_{s}^{*}\right\rangle$. There are two possible exceptions to this; the gamma distribution with parameters Ω_*IV*_ and the exponential with Ω_*FV*_, but even here, the correspondence is reasonably good. It would appear therefore that, for a given s-MSPRT, there is a u-MSPRT counterpart with the same mean decision sample and error rate. Further, given the provisos above, Eq 15 implies that decision times in the two cases will be equal (ignoring the residual term 0.5μ*) if we assign *δt* = μ*. In the Methods, we develop a heuristic argument to show why there is a close match between the two methods.

### s-MSPRT can be more observation efficient than the usual u-MSPRT

Consider Eq 4. Then, putting $R(T)=\sum_{k=1}^{N}\exp y_{k}(T)$, Eq 4 becomes

$$\begin{array}{c} P_{i}\left(T\right)=\frac{\exp y_{i}\left(T\right)}{R(T)} \end{array}$$ (16)

where, *R*(*T*) is a hypothesis independent normalisation constant. The idea, therefore, is to think of the posterior as a ‘scaled’ version of exp *y*_*i*_(*T*), although this scaling will change at every spike arrival in any channel. This is illustrated in Fig 2, in which panels b,c therein show *y*_*i*_(*T*) and *P*_*i*_(*T*) respectively; the notion of scaling is especially clear for the preferred hypothesis. Assuming a small error rate, most decisions will choose this hypothesis, and so the critical feature for the decision time is the trajectory of the integrated evidence therein, y_*_(*T*) (here, and henceforth, a ‘*’ subscript denotes quantities on the preferred hypothesis).

In general, the terms L_*_(*j*) in Eq 9 contributing to y_*_(*T*) have similar form whether they are notionally obtained from spike arrivals or uniform sampling. Therefore, at the level of observations, typical trajectories for P_*_(*T*) will be similar in u- and s-MSPRT, up to an overall scaling by *R*(*T*). However, even with identically shaped trajectories, the decision sample will depend on *R*(*T*) and the threshold in each case. We therefore proceed to examine these quantities.

In evaluating *R*(*T*), we first we note that each contributory term *L*_*i*_(*j*) to the evidence in the null hypothesis (in either MSPRT) is likely to be negative, since we expect f_*_(*x*_*i*_(*j*))/*f*_0_(*x*_*i*_(*j*)) < 1. This, in turn tends to make *y*_*i*_(*T*) < 0; as an example of this, see Fig 2b. Secondly, suppose we have taken the same number of observations in both u- and s-MSPRT on the preferred channel. Then, since μ* < *μ*_0_, there will be fewer observations in a null channel for s-MSPRT than for u-MSPRT because, for the former, they are sampled *μ*_0_/μ* times more slowly than those on the preferred channel, whereas all channels are sampled at the same rate for u-MSPRT. Incidentally, note that given the equality of decision samples (with respect to the preferred channel) this means that the total number of scalar observations, summed across all channels, to reach decision is less for s-MSPRT than it is for u-MSPRT. Thence, s-MSPRT is more *observation efficient* than its non-spiking counterpart.

### There is no single optimal ISI distribution for decision making

**Fig 5:**
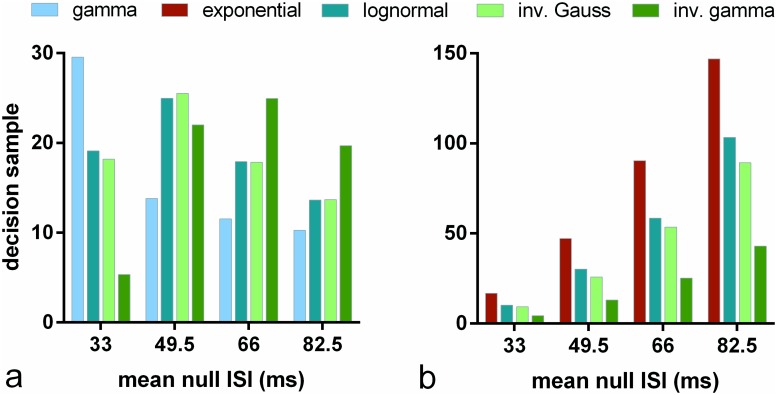
Decision sample in s-MSPRT against mean ISI for range of pdfs and parameter sets. Each bar shows, for the pdf indicated in the legend, the mean decision sample for *N* = 10 alternatives, averaged over 950 correct out of 1000 total trials. Panel a used parameter sets Ω_*IV*_, ${\hat{\Omega }}_{IV}(49.5)$, ${\hat{\Omega }}_{IV}(66)$, ${\hat{\Omega }}_{IV}(82.5)$, panel b used Ω_*FV*_, ${\hat{\Omega }}_{FV}(49.5)$, ${\hat{\Omega }}_{FV}(66)$, ${\hat{\Omega }}_{FV}(82.5)$ (see Methods). Each group of bars relates to one parameter set with its *μ*_0_ indicated on the *x* − axis (Ω_*IV*_, Ω_*FV*_ have *μ*_0_ = 33). For the case of Ω_*FV*_ and any ${\hat{\Omega }}_{FV}(\mu_{0})$, the gamma and exponential distributions are identical and so not reported separately.

There is a clear distinction in Fig 4 between the performance of algorithms assuming the different distributions. Is the rank ordering of performance maintained as we vary the distribution statistics? To explore this we repeated the experiments with s-MSPRT corresponding to *N* = 10 in Fig 4, but with other parameter sets ${\hat{\Omega }}_{IV}(\mu_{0})$, ${\hat{\Omega }}_{FV}(\mu_{0})$, *μ*_0_ = 49.5,66,82.5 derived from the original sets Ω_*IV*_, Ω_*FV*_ (see Methods). The results are shown in Fig 5, which also show those for Ω_*IV*_, Ω_*FV*_ for comparison (*μ*_0_ = 33 ms).

Fig 5a shows a clear difference in relative performance over the parameter sets Ω_*IV*_, ${\hat{\Omega }}_{IV}(\mu_{0})$ defined by their means. Further, there is some patterning to the variation in which the peak decision sample is a unimodal function of mean ISI. The inverse gamma shows best performance for small means, but is superseded by the other distributions at higher means, and in particular, by the gamma distribution. However, the latter is not a good fit to the data sets we examined (see Fig 10) and so a realistic mechanism might be more likely to use the lognormal or inverse Gaussian, both of which have similar decision samples.

In contrast, Fig 5b shows no such variation of performance for the sets Ω_*FV*_, ${\hat{\Omega }}_{FV}(\mu_{0})$, defined by their means and variances; the rank order is preserved. The inverse gamma also shows best performance with this parameterisation. In sum, there would appear to be no consistently ‘best’ distribution for decision making and that distribution contingent performance varies with the statistics of the data distributions.

Note also that the decision samples under the parameter sets ${\hat{\Omega }}_{FV}(\mu_{0})$ in Fig 5b are much larger in general than they are for those derived from Ω_*IV*_ in Fig 5a. This is because, for ${\hat{\Omega }}_{IV}(\mu_{0})$, the standard deviation for f_*_ and *f*_0_ does not grow with *μ*_0_, whereas it does for ${\hat{\Omega }}_{FV}(\mu_{0})$, since it is always equal to μ*, *μ*_0_.

### MSPRT is robust under variation of hypothetical distribution

**Fig 6:**
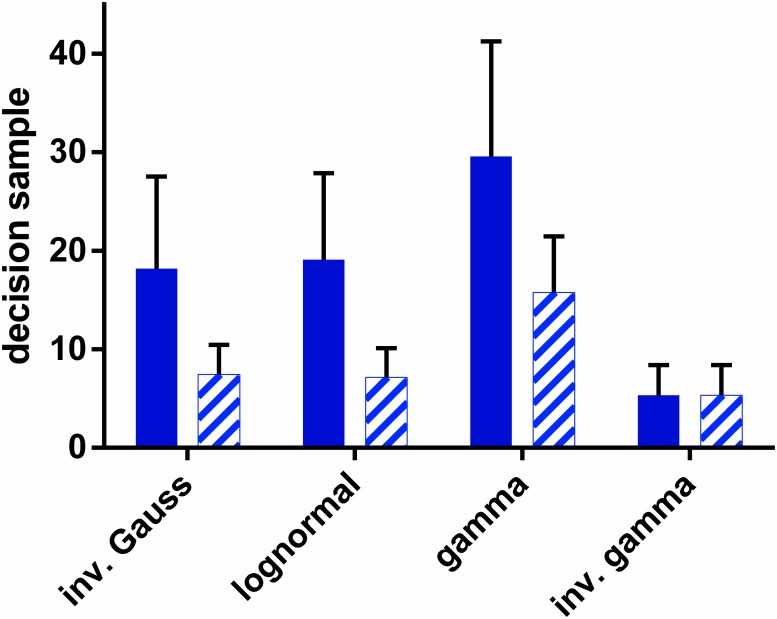
Decision sample for u-MSPRT when the distributions in the data were not matched to those tested for. Each bar shows the mean decision sample, for *N* = 10 alternatives, averaged over 950 correct out of 1000 total trials. The parameter set was Ω_*IV*_. The pale, patterned bars are for the case when the data is always sampled from an inverse gamma distribution, but inserted into mechanisms which test using the distribution indicated on the *x* − axis (by definition, the bars have equal height for the inverse gamma). The solid bars are for the case when the tested-for distribution matches the true distribution of ISIs, as indicated on the *x* − axis. Error bars are at one standard deviation.

Thus far we have assumed that the underlying statistics of the spike trains are the same as those of the hypothetical distributions (those in the likelihood). In particular, we have assumed that the functional form of the spike ISI distributions $f_{*}^{s}$, $f_{0}^{s}$ were identical to their hypothetical counterparts in the decision mechanism, $f_{*}^{h}$, $f_{0}^{h}$, (so that no distinguishing superscript *h*, *s* was required). However, in general, the decision mechanism may not ‘know’ *a priori* what form $f_{*}^{s}$, $f_{0}^{s}$ take. We now ask the question: what effect would an incorrect choice of the pdf form $f_{*}^{h}$, $f_{0}^{h}$ take on the decision time? To investigate this we fixed the distributions of ISIs to be inverse gamma, and supplied this data to decision mechanisms (u-MSPRT) based on a variety of pdfs. We used the Ω_*IV*_ parameter set for both distribution sets throughout. The results are shown in Fig 6 by the pattern filled bars. There is always a change in performance when an ‘incorrect’ hypothetical distribution form is used. However, for all the ‘incorrectly’ established mechanisms, the performance is better with the inverse-gamma-distributed data than that when each mechanism uses observations drawn from pdfs that are the same in the data and the hypothesis test (solid bars in Fig 6). Thus, there is no catastrophic decline in performance when using non-matching hypothetical distributions, and performance variation appears more intimately linked to the characteristics of the observations themselves.

### Expected total information gain for a decision is constant

It is clear from several of our results that the choice of pdf can make a substantial difference to the decision making performance. However, it is not clear what characteristics of the choice of pdf cause these differences. We might suppose that performance will be a function of how ‘far apart’ are the distributions f_*_, *f*_0_ and one popular measure of this distance is the Kullback-Leibler divergence (KLD) between two pdfs *p*(*x*), *q*(*x*) [33], defined by

$$\begin{array}{c} D\left(p||q\right)=\int_{-\infty }^{\infty }p\left(x\right)\log_{2}\frac{p(x)}{q(x)}dx \end{array}$$ (17)

Here we will use base-2 logarithms, so that results can be reported in bits. Note the KLD is, in general, asymmetric with *D*(*p*‖*q*) ≠ *D*(*q*‖*p*) (although symmetry may occur under some circumstances—*e.g.* Gaussians with different means and the same variance).

Now let *p* ≡ f_*_, *q* ≡ *f*_0_, then, taking expected values of the per-observation ratios in Eq 9 with respect to f_*_

$$\begin{array}{c} D(f_{*}\left|\right|f_{0})={\left\langle L_{i}\left(j\right)\right\rangle }_{f_{*}}\;\forall i,j \end{array}$$ (18)

The KLD is therefore the mean increase in log-likelihood, *y*_*i*_(*T*), per observation [41]. Denoting quantities on the preferred channel by *, and using Eq 9 together with Wald’s identity [42, 43], we can find the corresponding expectation of the accumulated evidence for the preferred hypothesis at decision time $T_{D}^{s}$,

$$\begin{array}{c} {\left\langle y_{*}\left(T_{D}^{s}\right)\right\rangle }_{f_{*}}=\left\langle s_{s}^{*}\right\rangle {\left\langle L_{*}\left(j\right)\right\rangle }_{f_{*}} \end{array}$$ (19)

Thus, using Eq 18

$$\begin{array}{c} \left\langle s_{s}^{*}\right\rangle =\frac{{\left\langle y_{*}\left(T_{D}^{s}\right)\right\rangle }_{f_{*}}}{D(f_{*}||f_{0})} \end{array}$$ (20)

**Fig 7:**
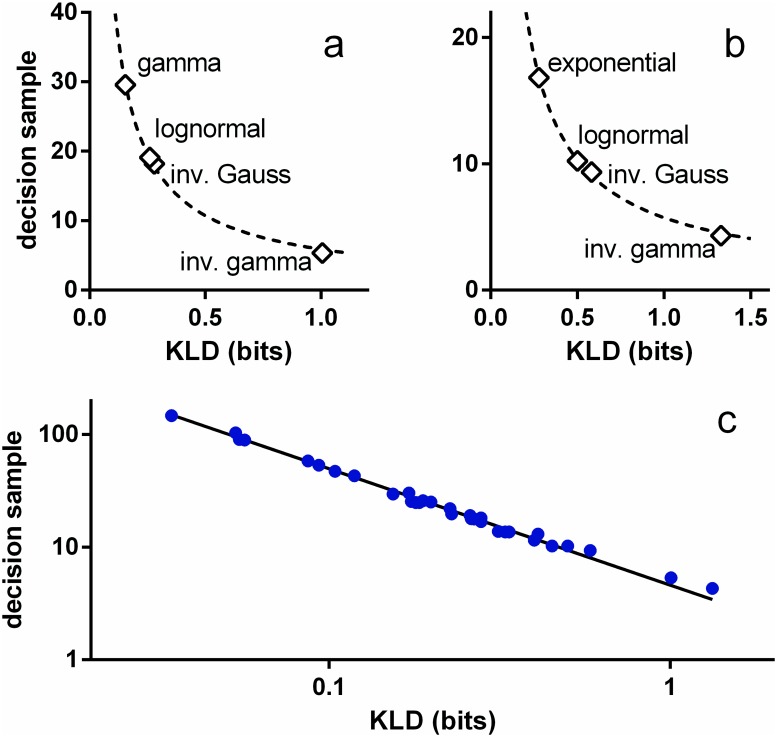
Mean decision sample for s-MSPRT against KLD. The KLD in all cases is *D*(f_*_‖*f*_0_) (see text). Panels a, b are for decision with the parameter sets Ω_*IV*_, Ω_*FV*_, respectively, and both use *N* = 10. The data points are the open symbols and the dashed lines, best fit power laws (nonlinear least squares). In panel c, the data points shown in blue symbols correspond to all the decision samples in Fig 5. The solid line is the best fit power law.

We now turn to an empirical investigation of Eq 20. If the numerator is constant for variation in parameters or pdf, we expect a simple inverse relation between $\left\langle s_{s}^{*}\right\rangle$ and *D*(f_*_‖*f*_0_). Fig 7a and 7b show that this is indeed true for each of the pdf classes Ω_*IV*_, Ω_*FV*_ (*μ*_0_ = 33 ms), used in Fig 5a and 5b respectively. The dashed lines are power law fits and are remarkably good: *R* ^2^ > 0.998, with exponents −0.866, −0.844, for Ω_*IV*_, Ω_*FV*_ respectively. These exponents are almost -1, as predicted by Eq 20 and, it would therefore appear that KLD is a good predictor of ‘local’ variations in performance under a given parameter set. However, plotting $\left\langle s_{s}^{*}\right\rangle$ against *D*(f_*_‖*f*_0_) for all 32 tests in Fig 5 indicates a more general result—see Fig 7c, where the log axes emphasise the power law fit over the wide range of the variables. The fitted function is

$$\begin{array}{c} \left\langle s_{s}^{*}\right\rangle =4.594D{\left(f_{*}||f_{0}\right)}^{-1.034} \end{array}$$ (21)

and again, the fit is very good (*R* ^2^ = 0.997). Just as significantly, the exponent is very close to -1, in which case the product of KLD and the decision sample is a constant *A*

$$\begin{array}{c} \left\langle s_{s}^{*}\right\rangle D(f_{*}\left|\right|f_{0})=A \end{array}$$ (22)

Some indication of why this might be true is supplied by Veeravalli and Baum [44] (see Methods). This indicates that the ‘constant’ *A* depends only on the error rate *ϵ* and number of hypotheses *N* according to

$$\begin{array}{c} A\left(\epsilon ,N\right)=(1-\epsilon -\frac{\epsilon }{N-1})\log\frac{\left(1-\epsilon )(N-1\right)}{\epsilon } \end{array}$$ (23)

(natural logarithm, note that in Fig 5, *ϵ* and *N* are fixed).

Further, Eq 22 also has theoretical plausibility grounded in the notion that KLD is a foundation of information theory. In fact, in its original formulation, it was noted that the KLD gave the mean *information per observation* for discrimination between two hypotheses about the distribution leading to the sample [33]. This kind of interpretation leads to the KLD also being referred to as an *information gain*. To emphasise this view we write *D*(f_*_‖*f*_0_) ≡ *I*(f_*_, *f*_0_). Then, using this, and Eq 23, the empirically supported result in Eq 22 becomes

$$\begin{array}{c} \left\langle s_{s}^{*}\right\rangle I\left(f_{*},f_{0}\right)=A\left(\epsilon ,N\right) \end{array}$$ (24)

The left hand side of Eq 24 is the product of the mean information gain per observation and the expected number of observations to decision; that is the mean total information required for the decision. Thus, Eq 24 states that, for a given *N* and *ϵ*, the mean total information for a decision is constant. In this view, the empirically determined constant *A* = 4.594 bits (Eq 21) is the expected amount of information required to perform a decision among 10 alternatives, with a 5% error rate, given uncertainty in the signals supplied. We now go on to use the result on information conservation to show how the neural refractory period can facilitate decision making and to explain two well known psychophysical phenomena.

#### The spike refractory period is a benefit, not an impediment, for decision making

In real biological neurons, spikes occupy a finite width and there is a refractory period between spikes which forces a lower limit on the ISI of not less than 1 ms. A popular choice for spike interval generation is the Poisson process for which the ISIs are distributed according to an exponential form *f*_*e*_(*x*). However, *f*_*e*_(*x*) has its mode (maximum) at *x* = *x*_mode_ = 0 thereby allowing the unrealistic occurrence of arbitrarily small ISIs. Nevertheless, if the assumption of *f*_*e*_(*x*) demonstrated a decision making procedure (*e.g.* s-MSPRT) with a performance advantage compared to other distributions, it could be that real neurons work to ensure their ISI distributions are as close to *f*_*e*_(*x*) as possible. On the other hand, if assuming *f*_*e*_(*x*) gives inferior performance compared to distributions for which *x*_mode_ > 0, the ostensible limitation in neural processing that is the refractory period may be thought of, instead, as a enhancing feature, since it is this mechanism that has forced a non-zero location of the mode.

To cast light on this issue, consider the results in Fig 4b. This shows a comparison of the performance of s- and u-MSPRT assuming *f*_*e*_ against a range of other distributions with *x*_mode_ > 0 using parameter set Ω_*FV*_. The procedures assuming the exponential distribution clearly give longer decision times compared to the others. This could be a peculiarity of the choice of the means, but examination of Fig 5b shows otherwise; in all cases the rank ordering of performance across distributions is maintained and the exponential performs worst. Similarly, the gamma assuming (u-)s-MSPRT performs worst among its cohort under Ω_*IV*_ (Fig 5b) while assuming distributions with maximum density at infinitesimal values of *x* (Fig 3b). By Eq 17, the KLD is determined by the ratio of the entirety of a pair of densities. When the modes of both are greater than 0 they also tend to differ from one another, as it happens in general in neural ISIs under preferred and null conditions, as well as in the rest of our densities under biologically realistic parameters (Fig 3a, 3c and 3d). Then, the area under the log-ratio of such densities and thus the KLD between them tend to increase which in turn, by Eq 24, improves the expected performance of a decision procedure. All this therefore supports the hypothesis that the existence of a refractory period helps neural decision making by facilitating the contrast of the ISI distributions of simultaneous spike trains.

#### Information conservation explains Hick’s Law

Hick’s law (sometimes known as the Hick-Hyman law) describes the relation often observed between mean reaction time *T*_*R*_ and the number of alternatives *N* in choice reaction time experiments with low error rates [35, 45, 46]. There are two slightly different variants

$$\begin{array}{ccc} T_{R} & = & a\log(N+1)\\ T_{R} & = & T_{0}+a\log\left(N\right) \end{array}$$ (25)

where the first form was that originally proposed by Hick and the second used in more recent applications [47]. The second form is also more plausible as the term *T*_0_ can absorb irreducible components of reaction time which originate in some minimum decision time, sensory processing delay and motor execution. In fact, in tasks where the stimulus-response mapping is too simple or over-learned (*e.g.* [48, 49]), the contribution of the logarithmic, *N* dependent factor appears to become undetectable [8].

The use of a log axis for *N* in Fig 4 emphasises the fit of the simulation data to laws of this form (all linear regressions of both kinds in Eq 25 have residuals *R* ^2^ > 0.95). Our results are therefore consistent with the psychophysics of choice experiments as expressed in Hick’s law.

Moreover, the information gain perspective is able to explain the general form of Hicks law. Starting from Eq 23, and assuming *ϵ*/(*N* − 1) to be negligible (due to small *ϵ*)

$$\begin{array}{ccc} \left\langle s_{s}^{*}\right\rangle I\left(f_{*},f_{0}\right) & \approx & \left(1-\epsilon \right)(\log\frac{\left(1-\epsilon \right)}{\epsilon }+\log\left(N-1\right))\\ & = & h(\epsilon )+(1-\epsilon )\log(N-1) \end{array}$$ (26)

with *h*(*ϵ*) = (1 − *ϵ*)log[(1 − *ϵ*)/*ϵ*]. For given neural distributions, *I*(f_*_, *f*_0_) is constant and so

$$\begin{array}{c} \left\langle s_{s}^{*}\right\rangle =a+b\log\left(N-1\right) \end{array}$$ (27)

which is close to the second form of Hicks law (in terms of a decision sample) given in Eq 25. Eq 27 is similar to the empirical expression for mean decision time of u-MSPRT obtained by McMillen & Holmes [8]. Through a different method, our expression completes their intuition, confirms the log(*N* − 1) dependence on *N* and demonstrates this form to generalize to s-MSPRT; both expressions constitute concrete, experimentally testable predictions.

**Fig 8:**
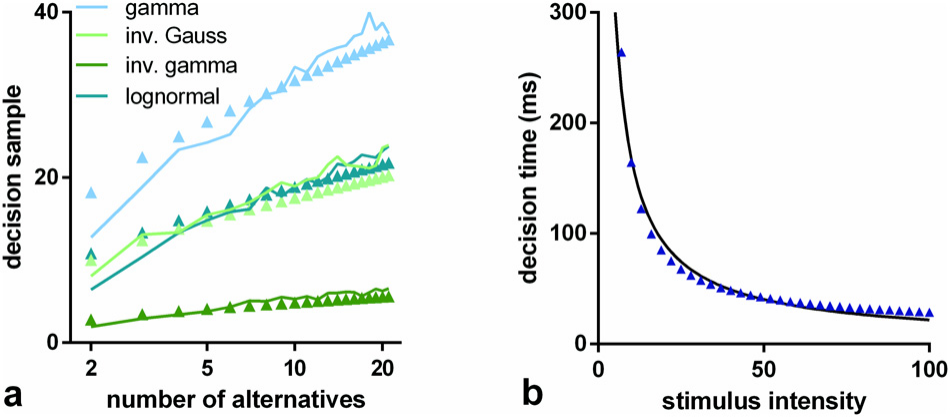
Hick’s and Piéron’s laws from conservation of information. Panel a is a direct counterpart of Fig 4a. The decision samples for s-MSPRT are shown as solid lines and the predictions from Eq 27 shown as solid symbols. Panel b shows results of the virtual experiment derived from Eq 30 (blue symbols) and a best fit power law (solid black line).

Further, we tested the ability of this theoretical result to quantitatively explain our data, by calculating *I*(f_*_, *f*_0_) for the distribution pairs used in Fig 4a, and substituted these in Eq 26; the results, together with the original simulation data are shown in Fig 8a There is good agreement with the empirical results.

#### Information conservation explains Piéron’s Law

There is another widely observed, lawful relationship in psychophysical experiments which has a long history. Piéron’s law [36, 50] is a psychophysical regularity where the simple reaction time to the detection of stimuli across a range of sensory modalities has been found to depend through a power law on the intensity of the stimulus, *u*. It is also a characteristic of low (≲ 5%) error rate circumstances [51, 52]. Piéron’s law has also been extended to the effect of stimulus intensity on choice reaction times (CRTs) [51, 53, 54] and to the effect of stimulus separability on CRTs [55]. In terms of the influence of stimulus intensity *u* on reaction time *T*_*R*_, Piéron’s law may be written as

$$\begin{array}{c} T_{R}=T_{0}+au^{-b},\;b>0 \end{array}$$ (28)

Just as in Eq 25, the term *T*_0_ may include components of reaction time which originate in minimum decision time, peripheral sensory delays and motor execution; we are interested only in the main decision making component *au* ^−*b*^.

To make the link between this component of Piéron’s law and Eq 24 we need a *linking hypothesis* which describes how neural responses, as firing rates, depend on *u*; that is we require firing rate *r* as a function of *u*. We base this on the study by Muniak et al on firing rates and tactile sensation [56]. Here, the task is one of stimulus detection and, while we are interested primarily in choices tasks, we take the kind of linking hypothesis developed in that study as indicative of a more general one. Further, we modified the relation *r*(*u*) used by Muniak et al [56] to include an explicit baseline firing rate *r*(*u*_*θ*_), where *u*_*θ*_ is the sensory threshold.

$$\begin{array}{c} r\left(u\right)=\alpha \log\frac{u}{u_{\theta }}+r\left(u_{\theta }\right) \end{array}$$ (29)

The use of a baseline ensures that we are comparing the firing in the preferred channel with a non-zero null rate; that is we assume *μ*_0_ = 1/*r*(*u*_*θ*_) for a fixed *f*_0_. Similarly, we assume the pdf of ISIs in the preferred channel, f_*_(*u*), is parameterised by its mean ISI μ*(*u*) = 1/*r*(*u*).

Then, starting from the decision time relation, $T_{D}(u)=\langle s_{s}^{*}\rangle \mu^{*}(u)$, and using the expression for $\left\langle s_{s}^{*}\right\rangle$ from Eq 24, we have

$$\begin{array}{c} T_{D}\left(u\right)=\frac{A(\epsilon ,N)}{[\alpha \log\frac{u}{u_{\theta }}+r\left(u_{\theta }\right)]D(f_{*}\left(u\right)||f_{0})} \end{array}$$ (30)

To test the validity of Eq 30 we ran a ‘virtual experiment’, obtaining simulated data points from Eq 30 with the following parameterisation, chosen to give firing rates comparable to those in the rest of this article: *α* = 10, *r*(*u*_*θ*_) = 10, *u*_*θ*_ = 1, 7 ≤ *u* ≤ 100; f_*_, *f*_0_ were lognormal with, *σ*_0_ = 200 ms, σ* = 65 ms; the error rate *ϵ* = 0.05, and *N* = 2. Fig 8b shows the resulting theoretical datapoints (dark blue symbols) and a best fit line of the form *T*_*D*_(*u*) = *au* ^−*b*^. The fit is good, and so it is possible for virtual experiments based on Eq 30—derived in turn from Eq 24—to be described by a Piéron-like law. We infer that Eq 24 with a linking relation like Eq 29 could account for Piéron’s law *in vivo*.

## Discussion

We have shown how arbitrary renewal spike trains may be subject to general Bayes-based sequential analysis and, when this treatment specialises to the use of log-probability ratios, we obtain an instance of the MSPRT. In this spiking MSPRT (s-MSPRT), the data are ISIs, and the sampling times are given by the asynchronous spike arrival times on several parallel channels. The corollary of this is that the posterior, for any hypothesis, is updated at spike arrivals on any channel.

Our analysis of ISI data for neurons involved in a decision task showed that this is not well approximated by a Gaussian. Indeed there is a conceptual problem that the Gaussian admits negative ISIs which is infeasible. On application of s-MSPRT to the most oft-used, realistic ISI distribution –the exponential yielding ‘Poisson spikes’– the accumulated evidence, *y*_*i*_(*T*), up to time *T*, is shown to be approximately equal to the scaled count of spikes to *T* (up to a scaling factor). This grounds previous work by Zhang and Bogacz (2010) [17] whose starting point was the *assumption* that the evidence in this case was given by a (scaled) spike-count. Our result also modifies the result of [17] showing that, while providing the main source of evidence, for an exponential-based s-MSPRT the spike count needs to be augmented by an additional ‘correction’ (*δ*_*i*_(*T*), in Eq 12).

Our data analysis also highlighted a series of candidate distributions which fitted the data better than the exponential (gamma, inverse gamma, lognormal, inverse Gaussian). The accumulated evidence for these was shown to be a spike count with additional terms which, in general, cannot be neglected.

We showed that s-MSPRT is comparable with a uniform sampling counterpart—u-MSPRT—if the sampling rate of the latter is set to the mean ISI on the preferred channel, μ*. However, in making this comparison, we argued that performance is best measured in most cases using the decision sample rather than decision time *per se* as the latter requires careful interpretation (see below). The decision samples for s- and u-MSPRT are very close and there is a heuristic argument to be made why this might be so. Nevertheless, s-MSPRT can achieve the same performance and error rate with fewer observations than u-MSPRT because it doesn’t have to accommodate as many observations from the non-preferred (‘null’) channels; thence, s-MSPRT is more observation efficient than u-MSPRT. As with many decision mechanisms (*e.g* [8, 10, 57, 58]), both s-MSPRT and u-MSPRT showed a Hick’s law relation between decision sample and number of alternatives.

A key result was that there is no universally optimal distribution for making a decision. The best performance varies with the underlying statistics of the data. However, a decision mechanism assuming the exponential distribution shows, in general, an inferior performance compared to when assuming other distributions with mode at non-zero ISI. Thus, the spiking refractory period can be viewed as positive feature which aids decision making mechanisms, rather than a drawback which hinders attainment of optimal performance therein.

In most of our simulations, the s-MPSRT was configured to give the best performance because the hypothetical distributions (*i.e.* those tested for) were matched to those of the data. That is, the decision mechanism was given ‘privileged’ information about the data. However, this will not normally be the case and we showed a case where this assumption is violated and there is no catastrophic decline in performance.

We showed empirically in our model that the mean total information gain required to make a decision was constant, over a wide range of distributions and parameters. This was supported by some analytic results and so we conjecture that it may be a quite general result. While this result may not sound surprising in isolation, it is remarkable that it emerges empirically in such a precise way from the examination of a variety of conditions with different distributions and parameterisations. This result allowed us to provide a theoretical rationale for Hick’s law and Piéron’s law and a concrete, experimentally testable expression for mean decision time as a function of sensory information content, error rate and number of choices (Eq 24).

### Interpretation of s-MSPRT and perceptual decision times

The grounding of a probabilistic decision mechanism in neural firing statistics is clearly a conceptual advantage, since neural decision mechanisms must ultimately make use of spikes in some way. However, we are confronted with interpretational issues which may not arise if we are working at a more abstract level of description, in which ‘evidence’ and ‘sampling times’ may be chosen to fit behavioural reaction times. First, assuming the brain performs decisions using a mechanism akin to s-MSPRT, we suppose it operates in neural systems which do not include sensory pre-processing (through early visual cortex, for example) or delivery of motor output (through motor cortex to spinal cord or brainstem). In reality, most probably these three stages are not sequential but overlap in time [59]; indeed, decision making may carry on during motor execution resulting in observable ‘changes of mind’ [60]. Nevertheless it is useful to conceive of these three contributions as separate processes as we are interested in the central, decision making stage, which will be observable electrophysiologically in studies like [30, 61–67].

Given this demarcation of processing, there is a wide range of possible neural decision times discernible in perceptual tasks. For example, using high contrast, easily distinguished stimuli in a paradigm designed specifically to tease out the perceptual decision making process, Stanford et al [68] showed that this could occur in as little as 30 ms for a 2-alternative task. However, for the relatively hard to distinguish stimuli in the random dot motion task (RDMT) with 2–4 alternatives, neural integration times can be several hundred ms [69, 70].

The statistics of the neural data we used here were obtained in a RDMT with stimulus coherence of 12.8% [30] (see Methods). In the RDMT-based study of Churchland et al (2008) [69], with stimulus coherence of 12.8%, the mean reaction times for *N* = 2 and *N* = 4 were 535 ms and 618 ms respectively. Using estimates of sensory and motor delays of 200–300 ms [60, 71] we would expect corresponding decision times of 285 ± 50ms, and 368 ± 50ms; that is of the order of 10^2^ ms. A full behavioural comparison with studies like this is out of the scope of the present article, however, if we convert the sampling times for small *N* in Fig 4a to decision times using Eq 15, we obtain decision times of the same order as this for several of the distributions, including the lognormal which gave the best fit to the data in [30, 31].

Thus far, we have predominantly used a simple version of s-MSPRT based on a single neural input stream on each channel (Fig 1b). In general, however, the theory (leading to Eq 10) is neutral regarding the origin of the spikes in each channel—they may derive from a single or multiple neural sources (Fig 1a). We now consider two possibilities for combining the information across them. In the first, we assume there is some mechanism of *spike superposition* to pool spikes across neurons into a unitary stream before being subject to s-MSPRT. In this case, for large numbers (∼ 100) of contributing streams with arbitrary distributions, the resulting superposition of spike trains has a distribution extremely close to the exponential (albeit with some subtleties of its power spectrum) [72]. If μ*, *μ*_0_ are the mean ISIs within each input stream for preferred and null channels respectively, then the *effective* means of the ISIs in the superposed streams in each channel for *M* inputs per channel are μ*/*M*, *μ*_0_/*M*. However, assuming a good approximation to the exponential distribution, the gain, $g_{0}^{E}=\log\;\lambda_{*}/\lambda_{0}$, remains unchanged, and so the accumulated evidence *y*_*i*_(*T*) is still a spike count which simply scales with *M*. This will render shorter decision times and lower error rates, as more information is available.

The second approach works by directly pooling observations over *M* inputs. It is obtained by directly extending the formalism leading to s-MSPRT to include more inputs than hypotheses (*C* > *N*). Thus, let $x_{k}^{r}(j)$ be the ISI from the *r* ^*th*^ spike train of channel set *k* at time $t_{k,r}^{j}$, and let $T_{k}^{r}=\max_{j}\{t_{k,r}^{j}:t_{k,r}^{j}\leq T\}$, then it is straightforward to show that the analogue of Eq 9 is

$$\begin{array}{c} y_{i}\left(T\right)=\sum_{r=1}^{M}\sum_{j=1}^{T_{i}^{r}}L_{i}^{r}\left(j\right),\;L_{i}^{r}\left(j\right)=\log\frac{f_{*}\left(x_{i}^{r}\left(j\right)\right)}{f_{0}\left(x_{i}^{r}\left(j\right)\right)}+B\left(T\right) \end{array}$$ (31)

Mechanistically, since the order of summation can be reversed, the operations of integrating likelihood ratio terms over time within a stream, and summing across contributory streams, can be conducted in either order. Since the individual ISI statistics are preserved in each stream, the individual $L_{i}^{r}(j)$ are distributed in the same way as their single stream (*M* = 1) counterparts. Using a slight abuse of the original terminology we refer to this method as *evidence oversampling*, as it is equivalent to a version of the original s-MSPRT which samples *M*-times faster than the rate given by the nominal ISI sampling times. We would therefore expect its decision times to scale, on average, as 1/*M*.

For large *M*, both spike superposition and evidence oversampling may comprise *dense coding* implementations (that is, actively engage a large fraction of the relevant neural population). *Sparse coding* ones could be devised by adding: (a) input spike trains that are weakly- or non-informative for the decision and (b) corresponding distributions in the likelihood functions which make weak contributions to them. All approaches in this study assume that spikes, and functions of spike statistics like $L_{i}^{r}(j)$, are independent over different inputs. This is almost certainly not the case. Studying the net effect of correlations on decision making remains an interesting problem since positive correlations among spike trains [73, 74] may be detrimental for discriminability, but negative serial ISI correlations may reduce the variability of the signal [75] and help on information transfer [76]. However, our simple independent-input cases, provide a starting point for future models of pooling evidence in these ways. Whatever the details of any ensuing models, the range of single neuron ISI statistics which can yield physiologically plausible decision times will be extended considerably by allowing combination or pooling over many inputs (*M* > 1).

### Optimality or sufficiency?

It is often assumed that the brain implements nearly-optimal decision making mechanisms. For *N* = 2, u-MSPRT collapses [29] to Wald’s sequential probability ratio test [77]. u-MSPRT is thence optimal in the sense that it minimizes the mean sample size (and decision time) to make decisions at any given error rate; for *N* > 2, it is asymptotically optimal as it minimizes it for vanishingly small error rates [10, 29].

Beck et al (2012) [32] have recently shown that decision mechanisms will, in general, be implemented in a fundamentally suboptimal way. The main reason they give for this is that the brain does not usually have access to the statistical structure of the task. This was precisely the issue addressed in the Results dealing with robustness. There, the true statistics of the stimuli were supplied by an inverse gamma distribution, but we used incorrect (non-inverse-gamma) hypothetical pdfs for the computation of probability ratios. This mismatch of hypothetical and real, underlying data distributions is inevitable; the real data will never comply exactly with an analytically tractable pdf. Such a mismatch may also occur when the response distribution of sensory neurons changes by learning or adaptation (as in [78–81]) and stops approximating ‘previously trained’ hypothetical distributions (although this seems not to happen in MT over RDMT training [82]).

The results in our experiment were that low decision samples were maintained and were, in all cases, lower than those of other mechanisms assuming non-inverse-gamma pdfs used in conjunction with their own, correctly matched data. In this case, therefore a deterministically sub-optimal decision mechanism (in the sense of Beck et al) *suffices*, because its potentially optimised variant (here with inverse gamma data matching the test-pdf) is better than its counterparts.

There is a related argument here which starts with the data. The dataset we used to establish the parameters Ω_*IV*_ was best fit by the lognormal, but MSPRT assuming this distribution does not have the best performance; this place is occupied by that assuming the inverse gamma. Thus, if the best performance (given the set of pdfs we used) requires data with an inverse gamma distribution (and ideally, inverse gamma hypothetical pdfs) why is the data not distributed in this way? We argue that, in general, neural codes will putatively result from a compromise in achieving several goals, including maximising discriminability of firing patterns, as discussed here, and expressing an efficient transformation of their inputs based on perceptual information [83–85].

### Neural substrates for s-MSPRT

While the s-MSPRT is grounded in the neural *communication* medium of action potentials (spikes) we have yet to address the way in which specific *computations* involved in mechanisms like s-MSPRT may be performed. Consider first the complete Bayes-based expression in Eq 4. This apparently complex form is subject to a mapping onto the basal ganglia—a subcortical group of interconnected nuclei involved in mediating action selection. Thus, Bogacz and Gurney (2007) [10] have shown that (at least simplified forms of) the basal ganglia architecture and cortex could implement Eq 4 when applied to a u-MSPRT, using inputs of Gaussian form. While the original MSPRT was not confined to Gaussian inputs [29], its expression in the form given in Eq 4 is essential for the mapping to basal ganglia-based architectures. Lepora and Gurney [19] have since extended this mapping to much wider range of decision processes (with arbitrary type, and numbers of, pdfs). Indeed there are several possible mappings from this form to basal ganglia and associated circuitry [10, 19, 86]. One possible substrate for a process like the s-MSPRT as a whole therefore, is the basal ganglia and its afferent and target structures.

In all of the anatomical mappings of MSPRT to basal ganglia, the cortex is the locus of the neural representation of integrated evidence *y*_*i*_(*T*). Moreover, it has often been assumed that the pdfs delivering input to these integrators are Gaussian (although this is not necessary [17, 19, 29, 37–39, 87]). The integration of Gaussian signals is straightforward if all Gaussian inputs have the same variance; it is simply the product of a gain and a term linear in the (abstract) signal input *L*_*i*_(*j*) = *x*_*i*_(*j*) [7, 10, 16]. However, the forms for *y*_*i*_(*T*) resulting from the distributions we consider here can be complex (see Table 1). We now offer some heuristic arguments to suggest how the accumulated evidence *y*_*i*_(*T*) might be computed in cortex. In order to ease notation, in what follows we drop indices on *x*_*i*_(*j*) and represent an ISI generically by *x*.

One term which occurs several times in defining the accumulated evidence is log *x*. Now, if *r* is the instantaneous firing rate with *r* = 1/*x*, then log *x* = −log *r*. The transfer function, from current *z* to firing rate *w*, of the simple leaky integrate and fire neuron (LIF) can, under the correct circumstances, approximate the form *w* = *log*(*z*) [88]. Now suppose that the input spikes with rate *r* give, after low-pass filtering by the membrane, a roughly constant current *z*. This will be proportional to *r*, as *z* = *cr*, and so we have *w* = *log*(*cr*) = *c* ^′^ + *log*(*r*), with *c* ^′^ = *log*(*c*). The constant terms like *c* ^′^ may be absorbed into the term *B*(*T*) in Eq 8. Thus if *r* was an input to an inhibitory LIF-like neuron, the neuron’s output could form an additive input −*log*(*r*) to a second neural stage which combines the required terms.

Now consider the computation of terms like (log *x*)^2^ which occur with the use of the lognormal distribution. Recall from the narrative immediately following Eq 11, that the sum of ISIs to decision time for the *i* ^*th*^ channel is just *T*_*i*_, and that this is approximately *T* for all *i*. Further, such constant terms makes no overall contribution to the outcome. Thus, a term linear in *x*, which gives rise to such a sum of ISIs, will have no effect on the decision time (within the approximation *T* = *T*_*i*_). We can, therefore, always add a term linear in x to any component with negligible effect on the decision outcome. Thus, we can replace the squared log term by (log *x*)^2^−*a* ^2^ *x* with constant *a*. Using *x* = 1/*r*, this becomes $\left(-\log\;r+a/\sqrt{r})(-\log\;r-a/\sqrt{r}\right)$. Each bracket contains a term in −log *r* which we can compute using the procedure described above. The term $a/\sqrt{r}$ involves division by $\sqrt{r}$ and could be achieved by shunting inhibition acting on a tonic firing rate *a* [89–91]. The multiplication of the brackets is also plausible using active dendritic processing [92, 93].

Finally, terms linear in *x* can (as noted previously) be ignored, and terms like 1/*x* correspond to additive input based on firing rate—often the default assumption for neural processing. In sum, all the computations required for *L*_*i*_(*j*) in Table 1 are available to neural machinery at the synaptic and circuit levels.

### From ISIs to behaviour

The s-MSPRT provides a direct link from the information in spike train ISIs to ‘neural decision times’ which are a component of an overall behavioural decision time or reaction time. Further this link is strengthened by the hypothesis, supported in our simulation work, that the mean total information gain required to make a decision is constant (for a given error rate and number of alternatives). This was a key to providing explanations for Hick’s and Piéron’s laws and provides a direct link from neural signalling to psychophysical observation. We argue that we have laid the foundations for a programme of work in which neural recordings and spike trains’ statistical analysis can be made the basis of predictions about behaviour in multi-alternative choice tasks.

## Methods

### Choosing probability distributions to model spike data

**Fig 9:**
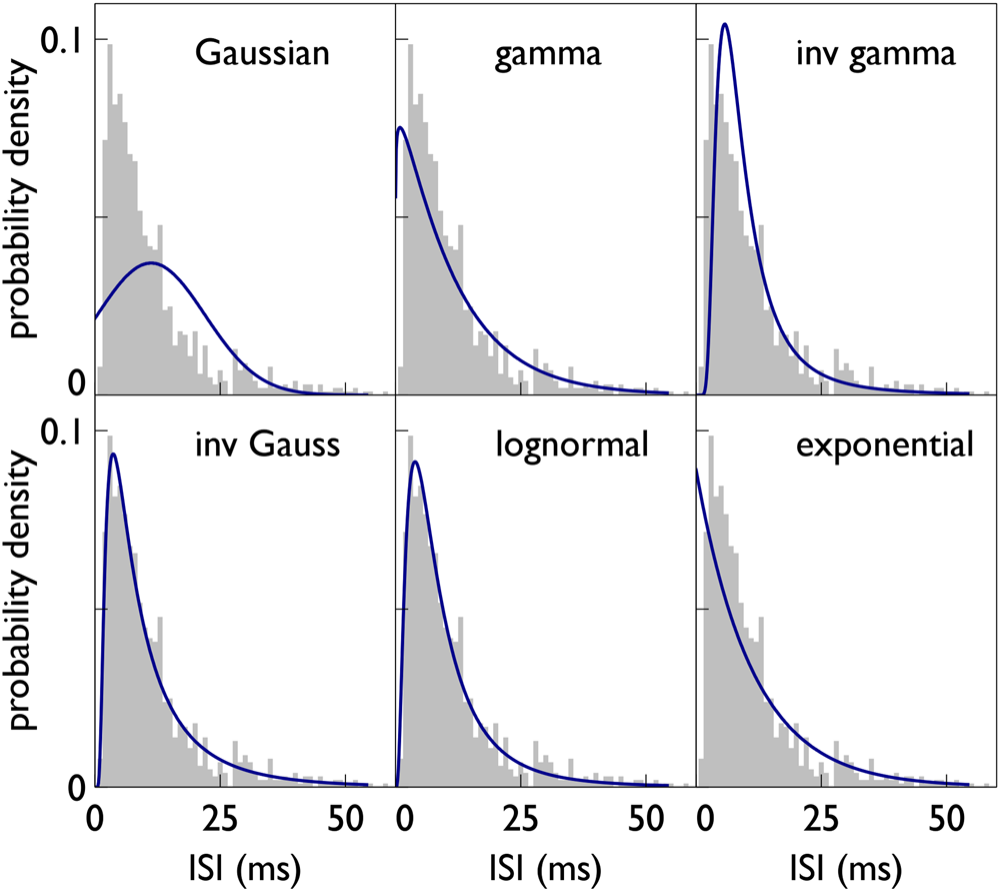
Fitting pdfs to an ISI histogram. The ISIs in the grey histogram (identical in each panel) were recorded by [31] from the MT neuron with tag e093 (Table 2). The few ISIs lying farther than four standard deviations beyond the mean were not plotted for clarity. Overlaid as a solid blue line in each panel is the best-fit pdf from a set of theoretical distributions: Gaussian, gamma, inverse gamma, inverse Gaussian, lognormal and exponential

Fig 9 shows the distribution of ISIs (grey histogram) in a macaque MT neuron, from the study by Britten et al [30, 31] using the RDMT. In this task, the monkey is typically shown two eccentric targets and then a kinematogram composed of dots in which a given percentage of them (the so-called ‘coherence’) move towards one of the targets, while the others move randomly. The animal is rewarded if it saccades towards this target from a gaze fixation point [30, 94]. The RDMT is therefore a perceptual decision making task of the kind we envisage being solved by a mechanism like the one proposed (s-MSPRT).

Each panel of Fig 9 shows the best fit (using the method of moments) of a pdf to the experimental data in the histogram. Data is assumed to come from single neurons and thus to be drawn from one such pdf; the implications for multiple neurons are considered further in the Discussion. All the pdfs are from the exponential family. The choice of putative distributions had the following rationale. The Gaussian, while an oft-used choice in more abstract (*e.g.* machine learning) approaches, cannot, in principle, be a genuine candidate for ISIs as there will always be a negative tail to the distribution which is physically implausible (there is no such thing as a ‘negative ISI’). However, it might be argued that this tail is, in most realistic cases, very small and that it can be neglected. We tested this possibility with real data sets (including that shown in Fig 9). The exponential distribution of ISIs leads to spike trains following a Poisson process—another popular choice for spike train analysis. This time, while the distribution satisfies the positivity requirement, it does not satisfy the requirement for excluding arbitrarily small ISIs due to a neural refractory period. In order to achieve this the distribution must have positive mode as well as lying wholly in the positive half-plane. In addition, we require that the pdf is, in general, positively skewed which matches the general shape observed in ISI data. These requirements are satisfied by suitable parameterisations of the other four candidate distributions: lognormal, gamma, inverse-Gaussian and inverse gamma.

**Fig 10:**
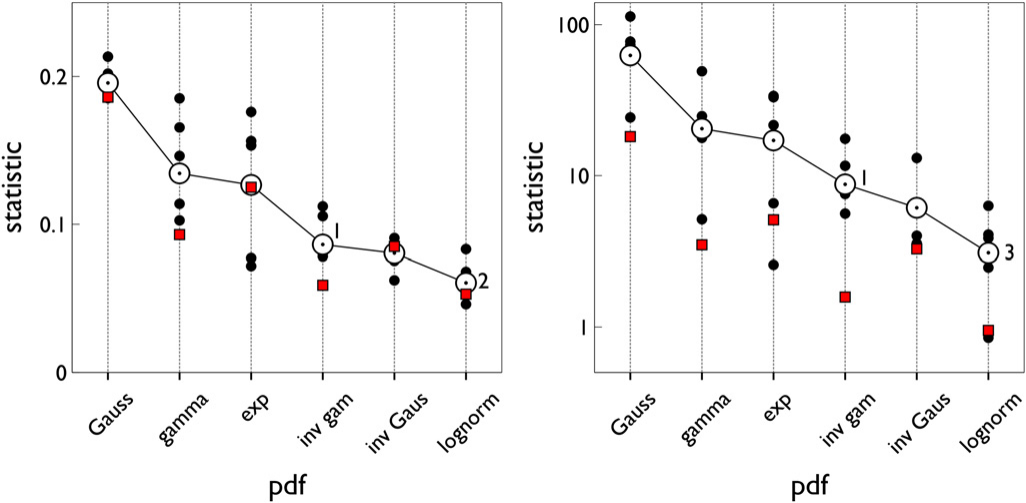
Goodness of fit of selected pdfs to ISI data. The black closed circles show the goodness of fit statistics for each of the first five data sets in Table 2. The red closed squares are for the data set from [27]. The mean for all six data sets is shown by the large open circles which also has a line plot; the number of significant fits at a level of 0.05 is noted per pdf next to such circles. Panels a and b are for the Kolomogorov-Smirnov, and Anderson-Darling tests respectively (note log y-axis in the latter). The ordering of the results from left to right preserves rank order of the mean statistic and is the same for both tests.

The inverse Gaussian and lognormal appear to be the best fit to the single data set in Fig 9. In contrast, note the large (implied) negative tail of the Gaussian, which could certainly not be neglected in using it to sample from for a decision process. However, aside from these qualitative observations, we sought to quantify the fitting process by repeating it with several data sets, and using two ‘goodness of fit’ metrics, the Kolmogorov-Smirnov and Anderson-Darling (see Fig 10) [95]. These tests are among the most powerful available for continuous and completely specified distributions [96]. The datasets are taken mainly from the Britten et al study [30] and are the first five described in Table 2. In order to avoid the transient due to stimulus onset, as advised by Bair [21] working with the same data set, we discarded the first 336 ms of all used spike trains. There is one other data set comprising recordings in the MT/middle-superior-temporal area, made during a paradigm using visual motion cues to initiate arm movements [27].

**Table 2:** Data sets from [31] used to generate ISI distributions.

Neuron tag	coherence	direction	number of trials	number of ISIs
j200	99.9%	1	7	1138
j200	6.4%	0	16	1030
e093*	25.6%	0	7	1044
e093	12.8%	1	6	1042
w059	51.2%	1	7	341
w144	12.8%	0	30	1360
w144	12.8%	1	30	2790

The Neuron tag is that used in the original data repository; the asterisk denotes the data used for the histogram in Fig 9. ‘Coherence’ is the percentage of the dots moving coherently in the random dot stimulus. ‘Direction’ (0 or 1) indicates overall stimulus direction: direction ‘1’ is the preferred direction of the neuron (across its entire tuning curve) while direction ‘0’ is its opposite. ‘Number of ISIs’ is the size of our sample, resulting from pooling data across the ‘number of trials’ indicated in the adjacent column.

Fitting results are in Fig 10, the smaller the statistic the better the fit. It is clear that the Gaussian is the worst fit to these data. Indeed, the scale on the Anderson-Darling plot is logarithmic largely to accommodate the Gaussian fits which have a mean of 62.5 compared to the next worst fit (the gamma) whose mean is 20.4. The exponential and gamma distribution had intermediate levels of fit, but were chosen for further study because of the useful comparison to be made with the oft-used exponential (giving Poisson statistics), and the fact that the gamma simplifies to the exponential when its mean and standard deviation are equal. The last three distributions (inverse gamma, lognormal and inverse-Gaussian) all have similar mean statistics, and are the best fits of all; in fact, it is remarkable that in a few instances (numbers next to the average-statistic symbols) the inverse gamma and lognormal pdfs significantly fitted the experimental data. In sum, we selected the gamma, inverse gamma, lognormal, inverse-Gaussian and exponential distributions for further study.

### Parameterising the distributions

For each selection of distribution we have to set four parameters: the mean μ* and standard deviation σ* of the data and test distributions f_*_ of the preferred channel or hypothesis (respectively), and their null counterparts *μ*_0_, *σ*_0_ for *f*_0_. We used two base-parameter sets, from which others were derived, The first base set we dub the *independent variance* parameter set, Ω_*IV*_, as both means and variances were independently fitted to one of the data sets from the RDMT study by Britten et al [30, 31]. Thus, μ*, σ* were fitted to the responses of neuron w144 when this was stimulated at coherence 12.8% with dots moving in its preferred direction (last row in Table 2). This neuron was singled out for being ‘typical’ of its parent population as its statistics closely matched a mean of the ISIs of over 40 MT neurons from [31]. The value of *μ*_0_, *σ*_0_ were obtained by fitting to the same neuron when stimulated at the same coherence but with motion in the null (anti-preferred) direction (sixth row in Table 2). This gave

$$\begin{array}{c} \Omega_{IV}=\;\left\{\mu^{*}=16.5,\;\sigma^{*}=21.5,\;\mu_{0}=33,\;\sigma_{0}=47.5\right\},\;\text{all}\;\text{units}\;\text{ms} \end{array}$$ (32)

The distributions resulting from using Ω_*IV*_ are shown in Fig 3a–3d. Other parameter sets (such as those in Fig 5a) are derived from Ω_*IV*_ by changing *μ*_0_, but keeping *μ*_0_ − μ* constant, and keeping the standard deviations of Ω_*IV*_. We will refer to these sets as ${\hat{\Omega }}_{IV}(\mu_{0})$

The exponential cannot be described by a set like Ω_*IV*_ as it only has a single parameter *λ* = 1/*μ*. In order to make comparisons with the exponential distribution, we therefore defined another *fixed variance* base-parameter set Ω_*FV*_

$$\begin{array}{c} \Omega_{FV}=\;\left\{\mu^{*}=16.5,\;\sigma^{*}=16.5,\;\mu_{0}=33,\;\sigma_{0}=33\right\},\;\text{all}\;\text{units}\;\text{ms} \end{array}$$ (33)

Other parameter sets (such as those in Fig 5b) are obtained by changing *μ*_0_, but keeping *μ*_0_ − μ* constant, and varying the standard deviations appropriately in order to maintain compatibility with the exponential. We refer to these sets as ${\hat{\Omega }}_{FV}(\mu_{0})$.

### Bounds on mean total information gain

Here, we give a plausibility argument for the empirical result in Eq 22. It is based on Lemma 2 in [44] which is framed in a more general situation where each hypothesis corresponds to a probability distribution *f*_*j*_. The Lemma makes uses the error probabilities

$$\begin{array}{c} \alpha_{k,l}=P(\text{decide}\;H_{l}\;\text{is}\;\text{true}|H_{k}\;\text{is}\;\text{true)} \end{array}$$ (34)

and states that, the expected number of observations ⟨*s*⟩_*k*_ to decision that any hypothesis *k* is true (with respect to a reference *j*), is given by

$$\begin{array}{c} {\left\langle s\right\rangle }_{k}\geq \frac{1}{D(f_{k}||f_{j})}\sum_{l=1}^{N}\alpha_{k,l}\log\frac{\alpha_{k,l}}{\alpha_{j,l}} \end{array}$$ (35)

In our situation, if *ϵ* is the error rate of the decision process,

$$\begin{array}{c} \alpha_{j,j}=1-\epsilon ,\;\alpha_{j,l}=\frac{\epsilon }{N-1},\;j\neq l \end{array}$$ (36)

so the sum on the right hand side of Eq 35 has only two non-zero terms

$$\begin{array}{ccc} \sum_{l=1}^{N}\alpha_{k,l}\log\frac{\alpha_{k,l}}{\alpha_{j,l}} & = & \left(1-\epsilon \right)\log\frac{\left(1-\epsilon )(N-1\right)}{\epsilon }+\frac{\epsilon }{N-1}\log\frac{\epsilon }{\left(1-\epsilon )(N-1\right)}\\ & = & (1-\epsilon -\frac{\epsilon }{N-1})\log\frac{\left(1-\epsilon )(N-1\right)}{\epsilon }\\ & \equiv & 𝓔(\epsilon ,N) \end{array}$$ (37)

Then, using our notation for the two distributions and for the expected number of observations to decision, Eq 35 for the preferred channel becomes

$$\begin{array}{c} \left\langle s_{s}^{*}\right\rangle \geq \frac{𝓔(\epsilon ,N)}{D(f_{*}||f_{0})} \end{array}$$ (38)

We now compare this result with that in Eq 20. Being an inequality, it might appear at first that Eq 38 is a weaker result than the equality in Eq 20. However, Eq 38 it is a strong result in so far as the numerator is explicitly independent of the distributions or their parameterisation. This makes it plausible that, in Eq 20, ${\langle y_{*}(T_{D}^{s})\rangle }_{f_{*}}$ is relatively constant. We therefore identify ℰ with *A* in Eq 22, so that *A* is now a function of *ϵ* and *N* with functional form given in Eq 37. Further, using the values of *N* and *ϵ* in our simulations, ℰ(0.05,10) = 4.856 which is only 5.7% different from the empirical value of 4.594 for the constant *A* in Eq 22. Taking these observations together, we conclude that, at least over the values of *N* and *ϵ* comparable with those used here, Eq 22 and Eq 24 have a plausible basis.

### A heuristic analysis of the relation between s-MSPRT and u-MSPRT

Returning to the analysis of *R*(*T*), let $\left\langle y_{0}^{u}(T)\rangle ,\langle y_{0}^{s}(T)\right\rangle$ be the expected value of the evidence on a null channel for u- and s-MSPRT, respectively. Then, using the arguments above, $\left\langle y_{0}^{u}(T)\right\rangle$ is formed from more observations than $\left\langle y_{0}^{s}(T)\right\rangle$, and their contributions are likely to be negative. Thus, $\left\langle y_{0}^{u}(T)\rangle <\langle y_{0}^{s}(T)\right\rangle$. Taking this result together with observation matching on the preferred hypothesis it is plausible that

$$\langle \text{exp}\;y_{0}^{u}\left(T\right)\rangle \begin{array}{c} <\langle \text{exp}\;y_{0}^{s}\left(T\right)\rangle ,\;\langle \text{exp}\;y_{*}^{u}\left(T\right)\rangle =\langle \text{exp}\;y_{*}^{s}\left(T\right)\rangle \end{array}$$ (39)

Let ⟨*R*_*u*_(*T*)⟩, ⟨*R*_*s*_(*T*)⟩ be the expected values of the normalisation terms in u- and s-MSPRT, where

$$\langle R_{u}\left(T\right)\rangle \begin{array}{c} =\langle \text{exp}\;y_{*}^{u}\left(T\right)\rangle +\left(N-1\right)\langle \text{exp}\;y_{0}^{u}\left(T\right)\rangle ,\;\;\langle R_{s}\left(T\right)\rangle =\langle \text{exp}\;y_{*}^{s}\left(T\right)\rangle +\left(N-1\right)\langle \text{exp}\;y_{0}^{s}\left(T\right)\rangle \end{array}$$ (40)

Then using Eq 39, ⟨*R*_*u*_(*T*)⟩ < ⟨*R*_*s*_(*T*)⟩. The expected value of the posterior P_*_(*T*) for u-MSPRT will therefore be larger at *T* than that for s-MSPRT. We conclude that, in order to preserve the observation count in the preferred channel before crossing threshold, we would expect the threshold *θ*_*u*_ for u-MSPRT to be greater than that for s-MSPRT, *θ*_*s*_. This result carries over to any monotonic function of the thresholds such as the exponential.

**Fig 11:**
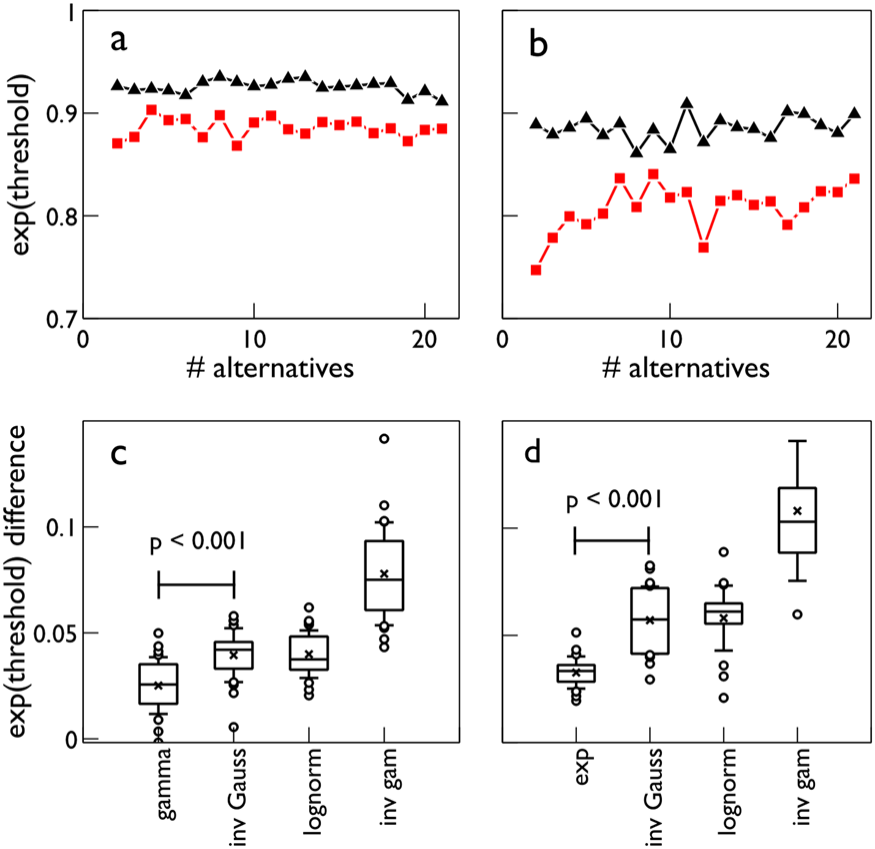
Decision thresholds and their difference between s-MSPRT and u-MSPRT. To ease visualisation, all panels show the exponential of the thresholds; *ϕ*_*s*_ = exp(*θ*_*s*_) *ϕ*_*u*_ = exp(*θ*_*u*_) for s- and u-MSPRT respectively. This yields positive values pertaining to the posterior (rather than negative values for the log-posterior). Panels a and b, respectively, show the lognormal and inverse Gaussian cases in Fig 4a for the parameter set Ω_*IV*_. The red lines and symbols are for *ϕ*_*s*_, the black lines and symbols for *ϕ*_*u*_. In panel c, the box plot labelled ‘lognormal’ shows the median and quartiles (box lines), mean (cross) and one standard deviation of the differences *ϕ*_*u*_ − *ϕ*_*s*_ in panels a and b. Other bars show similar quantities for the test distributions of the other MSPRT instantiations used to form Fig 4a. Panel d is similar to panel c, except it pertains to differences in (exponential) thresholds for results in Fig 4b, with the parameter set Ω_*FV*_.

Fig 11 shows that this condition on the thresholds is indeed met. Fig 11a and 11b show exp(*θ*_*s*_) < exp(*θ*_*u*_) consistently across all numbers of choices for two examples (lognormal and inverse Gaussian based s-MSPRT) for parameter set Ω_*IV*_. (The lognormal is more typical with little variation with the number of alternatives). Similar clear differences Δ_*e*_ *θ* = exp(*θ*_*u*_) − exp(*θ*_*s*_) exist for all distributions across both parameter sets Ω_*IV*_, Ω_*FV*_—see Fig 11c and 11d. Notice that the gamma distribution in Fig 11c and the exponential distribution in Fig 11d have the smallest Δ_*e*_ *θ* in their groups (and in each case, Δ_*e*_ *θ* is significantly less than the next smallest one at *p* < 0.001, two-sided t-test). This may account for the fact that decision times for the gamma distribution (Fig 4a) and the exponential distribution (Fig 4b) fail to achieve the criterion of equal decision sample as well as the others.
